# An oxindole efflux inhibitor potentiates azoles and impairs virulence in the fungal pathogen *Candida auris*

**DOI:** 10.1038/s41467-020-20183-3

**Published:** 2020-12-22

**Authors:** Kali R. Iyer, Kaddy Camara, Martin Daniel-Ivad, Richard Trilles, Sheila M. Pimentel-Elardo, Jen L. Fossen, Karen Marchillo, Zhongle Liu, Shakti Singh, José F. Muñoz, Sang Hu Kim, John A. Porco, Christina A. Cuomo, Noelle S. Williams, Ashraf S. Ibrahim, John E. Edwards, David R. Andes, Justin R. Nodwell, Lauren E. Brown, Luke Whitesell, Nicole Robbins, Leah E. Cowen

**Affiliations:** 1 Department of Molecular Genetics, University of Toronto, Toronto, ON Canada; 2 Department of Chemistry and Center for Molecular Discovery (BU-CMD), Boston University, Boston, MA USA; 3 Clark+Elbing LLP, Boston, MA USA; 4 Department of Biochemistry, University of Toronto, Toronto, ON Canada; 5 Department of Medicine, University of Wisconsin School of Medicine and Public Health, Madison, WI USA; 6 Department of Medical Microbiology and Immunology, University of Wisconsin, Madison, WI USA; 7 Division of Infectious Disease, The Lundquist Institute for Biomedical Innovation Los Angeles Biomedical Research Institute at Harbor-University of California, Los Angeles (UCLA) Medical Center, Torrance, CA USA; 8 Infectious Disease and Microbiome Program, Broad Institute of MIT and Harvard, Cambridge, MA USA; 9 Department of Biochemistry, University of Texas Southwestern Medical School, Dallas, TX USA; 10 David Geffen School of Medicine, UCLA, Los Angeles, CA USA

**Keywords:** Chemical biology, Antifungal agents, Fungi, Pathogens

## Abstract

*Candida auris* is an emerging fungal pathogen that exhibits resistance to multiple drugs, including the most commonly prescribed antifungal, fluconazole. Here, we use a combinatorial screening approach to identify a *bis*-benzodioxolylindolinone (azoffluxin) that synergizes with fluconazole against *C. auris*. Azoffluxin enhances fluconazole activity through the inhibition of efflux pump Cdr1, thus increasing intracellular fluconazole levels. This activity is conserved across most *C. auris* clades, with the exception of clade III. Azoffluxin also inhibits efflux in highly azole-resistant strains of *Candida albicans*, another human fungal pathogen, increasing their susceptibility to fluconazole. Furthermore, azoffluxin enhances fluconazole activity in mice infected with *C. auris*, reducing fungal burden. Our findings suggest that pharmacologically targeting Cdr1 in combination with azoles may be an effective strategy to control infection caused by azole-resistant isolates of *C. auris*.

## Abstract (web summary)

The fungal pathogen *Candida auris* is resistant to multiple drugs including the common antifungal fluconazole. Here, Iyer et al. identify a compound that potentiates fluconazole activity against *C. auris* in vitro and in vivo by inhibiting a major efflux pump and thus increasing intracellular fluconazole accumulation.

## Introduction

The rise in antimicrobial resistance has become a major threat to public health^1^. Although the focus has primarily been on pan-resistant bacteria, there is growing concern over the multidrug-resistant fungal pathogen, *Candida auris*. This emerging pathogen has galvanized researchers, health care workers, and the media due to its high rates of drug resistance and transmissibility^2,3^. In its most recent report, the U.S. Centers for Disease Control and Prevention classified *C. auris* as one of only five pathogens that are the most urgent threat to public health^4^. Thus, the emergence of *C. auris* highlights the need for more therapeutic options to combat drug-resistant fungal infections.

*C. auris* has an interesting history. Since it was first identified in 2009 in Japan^5^, genomic analyses have revealed the near simultaneous emergence of distinct lineages across six continents, encompassing over 30 countries within the past ~400 years^6,7^. Currently, the majority of *C. auris* isolates fall into four major geographical clades: South Asian (I), East Asian (II), African (III), and South American (IV)^6,7^. This species has a remarkable ability to persist on human skin and other surfaces for extended periods of time, which facilitates hospital transmission amongst patients who are already vulnerable to infection^2,8,9^. Additionally, the prevalence of drug resistance amongst *C. auris* isolates is widespread as recent studies show that over 80% of clinical isolates are resistant to the azole antifungal fluconazole^3,7^. Resistance levels vary significantly between clades, with some isolates exhibiting resistance to all three major antifungal drug classes available to treat systemic infections^3,7^.

The prevalence of fluconazole resistance amongst *C. auris* isolates is challenging from a clinical perspective as fluconazole is the most widely administered antifungal. This is due to its oral bioavailability, broad spectrum of activity, and favorable safety profile^10^. Fluconazole inhibits the biosynthesis of ergosterol, the major sterol in fungal cell membranes, through inhibition of lanosterol demethylase, which is encoded by *ERG11*^11^. Inhibition leads to an increase in the Erg11 substrate lanosterol, and the production of aberrant sterol intermediates, including 14-α-methyl-3,6-diol^12,13^. Mechanisms of fluconazole resistance amongst *C. auris* isolates are highly variable and often clade specific, the nuances of which are still being elucidated. One major mechanism of fluconazole resistance involves point mutations in hot spot regions in its target gene *ERG11*, which are known to confer resistance in other fungi^14–16^. In addition to *ERG11* mutations that are shared across all clades^3,7^, the most common substitutions found in clade I and IV are Erg11^Y132F^ or Erg11^K143R^, whilst clade III isolates commonly have an Erg11^F126L^ substitution. Notably, strains from clade II generally have no specific *ERG11* mutations and include the most sensitive isolates^3,6,16^. In addition to target alteration, *C. auris* encodes an array of multidrug transporters, several of which are strongly induced under various conditions, including fluconazole treatment^17–20^. Finally, *C. auris* isolates possess other genetic alterations that could confer fluconazole resistance, such as gene duplication leading to a higher copy number of *ERG11*^18^, or transcriptional upregulation of efflux pumps through mutations in *TAC1B*^21^. Overall, the diversity of *C. auris* resistance mechanisms is extensive, and the prevalence of fluconazole resistance threatens to render this important therapeutic obsolete in treatment of the rising number of *C. auris* infections world-wide.

A well-established strategy to thwart drug resistance and restore antimicrobial efficacy is the use of combination therapy, which has been successfully implemented for many difficult to treat infections, including HIV-AIDS, tuberculosis, and malaria^10^. By identifying agents that re-sensitize pathogens to existing therapeutics, the lifespan of existing antifungals could be extended. In vitro data suggests combining existing antifungals can be effective against *C. auris*^22,23^. An excellent example of the potential value of combination therapy is provided by iKIX1, a novel compound that inhibits interaction of the transcription factor Pdr1 with the Mediator complex in the fungal pathogen *Candida glabrata*, thus preventing upregulation of the multidrug transporter Pdr5^24^. Combination treatment with iKIX1 and fluconazole abrogated intrinsic azole resistance and improved survival in a murine model of *C. glabrata* infection^24^. Clearly, the inclusion of agents capable of impairing the most common, readily anticipated modes of antifungal resistance provides a rational, readily implemented strategy in the development of more efficacious combination treatment regimens.

In this study, we applied a combinatorial approach to screening of a chemically diverse library against an azole-resistant strain of *C. auris* to identify molecules that specifically enhanced the activity of fluconazole. We identified azoffluxin as a compound that synergized with fluconazole by increasing intracellular fluconazole accumulation through inhibition of the major multidrug efflux transporter Cdr1. Using azoffluxin as a chemical probe, we established that efflux is a major mechanism of resistance in isolates belonging to three of the four major *C. auris* clades. Notably, clade III isolates carrying specific mutations in *ERG11*, in addition to upregulating the multi-drug transporter Mdr1, remained resistant to fluconazole in the presence of azoffluxin, despite the compound blocking efflux of Nile red and fluconazole in these isolates. Azoffluxin showed cross-species activity by potentiating fluconazole activity against a resistant isolate of *Candida albicans*, the most common human fungal pathogen^25^. In culture, azoffluxin transformed fluconazole from ineffective to highly active in rescuing mammalian cells infected with drug-resistant *C. auris*. In mice infected with drug-resistant *C. auris*, azoffluxin not only enhanced fluconazole activity but also reduced fungal burden by ~1000-fold as a single agent. Collectively, our findings demonstrate pharmacological inhibition of Cdr1 function may be an effective way to control infection with azole-resistant isolates of *C. auris*.

## Results

### Chemical screen identifies azole-synergizing compound

**Fig. 1:**
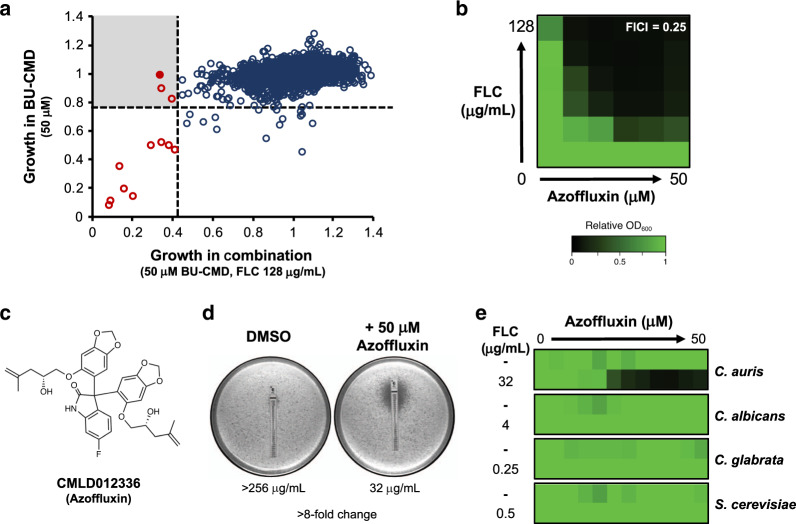
Screen of the BU-CMD library identifies azoffluxin as a fluconazole (FLC) potentiator against *C. auris*. **a** BU-CMD library was screened at 50 μM in the presence or absence of 128 μg/mL of FLC in RPMI medium at 30 °C for 48 h. Growth of *C. auris* strain VPCI 673/P/12, as determined by optical density at 600 nm (OD_600_), is plotted in the presence of each CMD compound alone and in combination with FLC. Dotted lines represent 7-median absolute deviations from the median for each condition. Red circles indicate compounds that showed significant bioactivity. The shaded quadrant indicates compounds that show enhanced activity in the presence of fluconazole, with azoffluxin shown as a filled red circle. **b** Checkerboard assays with azoffluxin (CMLD012336) and FLC were performed in RPMI at 30 °C by titering 2-fold dilution series of azoffluxin and FLC. Growth in each well is presented in heat-map format based on the OD_600_ of wells at 48 h relative to the no-drug control (see color bar). The fractional inhibitory concentration index (FICI) was calculated to assess interaction effect, with a value <0.5 indicating synergy. **c** Structure of CMLD012336 (azoffluxin). **d** FLC Etest strips in the presence and absence of 50 μM azoffluxin. *C. auris* cells (1 × 10^6^) *C. auris* cells were plated on YPD agar, the E-test strip was added, and plates were incubated at 30 °C for 24 h prior to imaging. **e** Dose-response assay based on 2-fold serial dilution of azoffluxin starting from 50 μM in the absence and presence of indicated concentration of FLC for *C. auris* Ci6684 (Erg11^Y132F^)*, C. albicans* (SN95)*, C. glabrata* (BG2), or *S. cerevisiae* (BY4741), respectively. The highest FLC concentrations that did not affect growth alone for each species was used. Dose-response assays were incubated for 48 h at 30 °C in RPMI. Growth in each well was quantified by the OD_600_ of treated wells relative to the respective no-drug control (see color bar in **b**). Source data are provided as a Source Data file.

To identify novel compounds that enhance the activity of fluconazole against *C. auris*, we screened a diversity-oriented, synthetic library created by Boston University’s Center for Molecular Discovery (BU-CMD). This library of 2454 molecules, many natural product-inspired, has been curated to encompass greater structural complexity than conventional chemical libraries^26^, which is a feature that increases the likelihood of identifying compounds with bioactivity against microorganisms^27,28^. The BU-CMD library was screened at 50 μM in the absence or presence of a concentration of fluconazole that inhibited growth of the fluconazole-resistant clade I *C. auris* strain VPCI 673/P/12 by ~20%. Through Sanger sequencing we confirmed this strain harbored both an Erg11^K143R^ substitution and Tac1b^A640V^ substitution. Compounds that reduced growth after 48 h compared to the control by 7-median absolute deviations from the median alone were classified as single agent antifungals; their mechanism of action has been described elsewhere^29^. Compounds for which antifungal activity was only observed in combination with fluconazole were classified as fluconazole potentiators (Fig. 1a). Of the three fluconazole potentiators identified, we prioritized the *bis*-benzodioxolylindolinone CMLD012336, a 3,3-diarylated oxindole hereafter referred to as azoffluxin, due to its strong synergistic interaction with fluconazole against a resistant strain of *C. auris*, Ci6684 (Fig. 1b, c). Ci6684 harbors an Erg11^Y132F^ substitution but no known activating substitutions in Tac1b (Tac1b^WT^). The fractional inhibitory concentration index (FICI) calculated for the combination was 0.25, with values <0.5 indicating a synergistic interaction (Fig. 1b)^30^. As a complementary approach, we used fluconazole E-test strips to determine whether synergy could also be observed on solid medium. In the absence of azoffluxin, *C. auris* grew up to the highest concentration of fluconazole present on the E-test strip. Strikingly, the presence of azoffluxin (50 μM) reduced the fluconazole minimum inhibitory concentration (MIC) >8-fold, from >256 μg/mL to 32 μg/mL on YPD agar (Figs. 1d and S1a). Finally, given the potent synergy against *C. auris*, we tested fluconazole-sensitive laboratory strains of *C. albicans* (SN95), *C. glabrata* (BG2), and *Saccharomyces cerevisiae* (BY4741), to represent diverse fungi. Interestingly, azoffluxin did not enhance the activity of fluconazole against any of these species even in the presence of the highest concentration of fluconazole that did not impair growth in each species on its own (Fig. 1e). Thus, either azoffluxin exerts species-selective activity or it only enhances fluconazole activity in the context of pre-existing resistance, but not in fluconazole-sensitive organisms.

### Azoffluxin enhances azole efficacy in a Cdr1-dependent manner

**Fig. 2:**
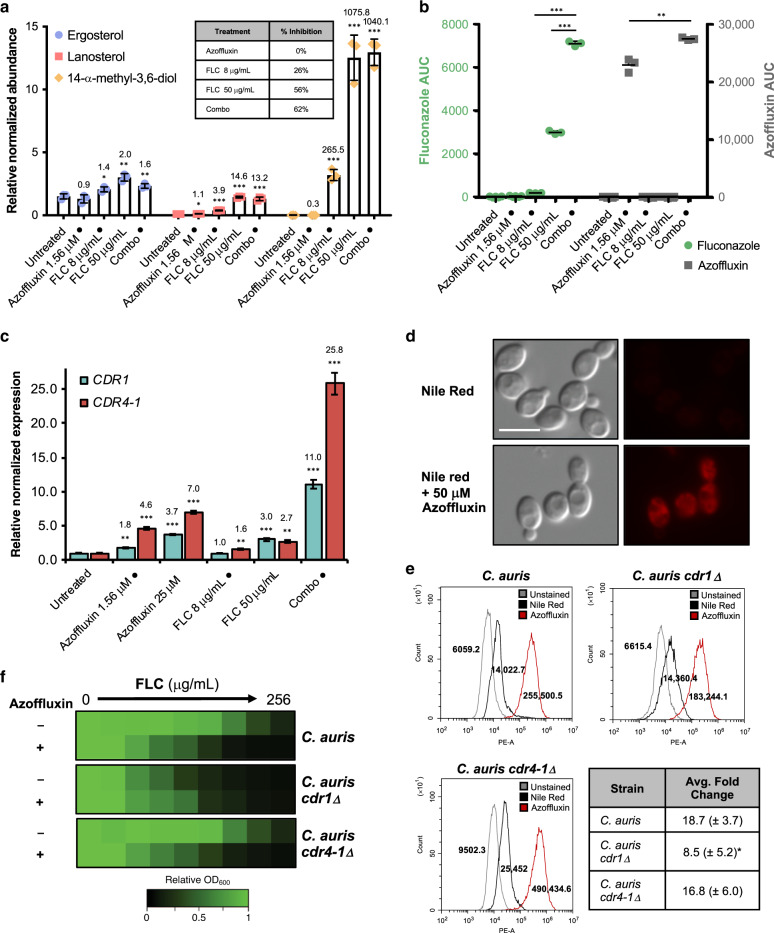
Azoffluxin increases intracellular accumulation of fluconazole (FLC) by inhibiting Cdr1-mediated efflux in *C. auris*. **a** Abundance of ergosterol (blue), lanosterol (red), and 14-α-methyl-3,6-diol (yellow) was determined in Ci6684 after compound treatment (• indicates concentrations used in combination treatment) relative to internal cholesterol standard. Growth inhibition (%) caused by each treatment is presented in table. Data are presented as mean ± SD of technical triplicates. Significance was determined by two-sided unpaired Student’s *t* test of condition compared to untreated; *p-value < 0.05, **p-value < 0.01. Fold-change for each treatment is indicated above the respective bar. **b** Intracellular concentrations of FLC (green) and azoffluxin (gray) were measured after treatment for 1 h. Data are presented as mean ± SD of technical triplicates. Significance was determined by two-sided unpaired Student’s *t* test, **p-value = 0.003, and ***p-value > 0.001. **c** Transcript levels of Ci6684 *CDR1* (teal) and *CDR4-1* (red) were measured. Cells were treated with indicated concentrations of compound (• indicates concentrations used in combination treatment). Transcript levels were normalized to *ACT1* and *GPD1* and are relative to the untreated control. Data are presented as mean ± SEM of technical triplicates. Significance of differences between untreated control and treatment was determined by two-sided unpaired Student’s *t* test; *p-value < 0.05, **p-value < 0.01, ***p-value < 0.001. Fold-change is indicated above each bar. **d** Ci6684 was treated with azoffluxin, followed by addition of Nile red. Scale bar represents 5 µm. **e** Cells from Fig. 2d and Fig. S2 were analyzed by flow cytometry. Histograms depict relative fluorescence intensity (PE-A) of events, values depict median fluorescence intensity (MFI). Table displays mean fold-change in MFI of azoffluxin-treated, Nile red stained cells ± SD for biological triplicates. Significance of difference determined by a two-sided unpaired Student’s *t* test, *p-value < 0.05 compared to parental average MFI. **f** Dose-response assays were conducted as in Fig. 1e. FLC was applied as a 2-fold dilution series in the absence or presence of azoffluxin (50 µM). Growth was monitored and normalized to no-drug control (see color bar). Source data are provided as a Source Data file.

Of the many ways in which drug combinations can exert a synergistic effect, a common mechanism involves one compound enhancing the biological effect of another agent by targeting parallel pathways or improving bioavailability^31^. To investigate these possibilities for azoffluxin, we profiled the sterol composition of *C. auris* with or without prior exposure to compounds^13^. Our hypothesis was that if azoffluxin heightens the effects of fluconazole-mediated Erg11 inhibition, a low concentration of fluconazole combined with azoffluxin would have an equally profound impact on sterol composition as a high concentration of fluconazole alone. Using LC-MS, we evaluated how exposure of *C. auris* to a combination of azoffluxin and fluconazole for 18 h affected the abundance of three membrane sterols: ergosterol, lanosterol, and the azole-induced aberrant sterol intermediate 14-α-methyl-3,6-diol (Fig. 2a)^12^. Minimal changes in abundance of these sterols were detected between untreated and azoffluxin-treated cells. Compared to untreated cells, fluconazole treatment resulted in significant 1.4-fold and 3.9-fold increases in ergosterol and lanosterol, respectively, and a larger >250-fold increase in 14-α-methyl-3,6-diol (*p* < 0.05; Fig. 2a). This suggested that while exerting a minimal effect on growth, the low fluconazole concentration partially inhibited Erg11, causing a compensatory upregulation in ergosterol biosynthesis. However, azoffluxin dramatically amplified the impact of the low fluconazole concentration on sterol composition. Most notable was a >1000-fold increase in 14-α-methyl-3,6-diol compared to untreated cells (Fig. 2a). The relative abundance of all three sterols upon treatment with both azoffluxin and a low concentration of fluconazole was similar to that seen in the sterol profile upon treatment of cells with a higher concentration of fluconazole alone, treatments that both resulted in ~50% growth inhibition (Fig. 2a).

To determine whether azoffluxin enhances the effect of fluconazole treatment by increasing intracellular fluconazole abundance, we measured intracellular levels of fluconazole using LC-MS after 1 h of treatment. We detected significantly more intracellular fluconazole in the combination treatment group compared to treatment with either a high or low fluconazole concentration alone (*p* < 0.001; Fig. 2b). This amounted to a ~2.5-fold increase in intracellular fluconazole in the combination treatment compared to high fluconazole, despite the fact that these treatments result in similar changes to the sterol profile of the cell (Fig. 2a). This discrepancy is likely due to the variant time points at which these assays were performed. Furthermore, we were able to detect intracellular levels of azoffluxin, which were increased in the combination treatment relative to azoffluxin alone. These results indicate that this compound acts intracellularly and fluconazole enhances its accumulation, perhaps due to the disruption of membrane homeostasis by azoles (Fig. 2b). Overall, our LC-MS profiles suggest that azoffluxin synergizes with fluconazole by increasing the intracellular accumulation of fluconazole through an undetermined mechanism that next we sought to define.

In pursuing mechanistic studies, we reasoned that an increase in intracellular azole accumulation could be caused either by enhancing permeability or by impeding drug efflux. To discriminate between these two models, we first tested the hypothesis that the increase in intracellular fluconazole caused by azoffluxin treatment resulted from impairment of multidrug efflux transporter activity. Impairment could be achieved by either a transcriptional mechanism that reduces the expression of genes encoding transporters or through a post-transcriptional mechanism. In order to evaluate potential transcriptional effects, we profiled the relative expression of six putative *C. auris* efflux genes, identified by Muñoz and coworkers^18^, following treatment with azoffluxin, fluconazole, or a combination of the two compounds (Fig. S1b). Of the six transporter genes assessed, those encoding the putative ABC transporters Cdr1 (B9J08_000164) and Cdr4-1 (B9J08_000479) demonstrated similar expression profiles. At exposures to azoffluxin alone, which had no effect on growth, we saw a concentration-dependent increase in both *CDR1* and *CDR4-1* transcript levels, which was greater than the induction observed upon treatment with fluconazole (Figs. 2c and S1b). Furthermore, we observed a greater increase in *CDR1* and *CDR4*-*1* expression upon combination treatment than the increase seen with any individual compound treatment (Fig. 2c). The observation that azoffluxin causes an increase in transcript level of two efflux genes but increases intracellular accumulation of fluconazole, suggested a model in which azoffluxin directly inhibits efflux transporter function post-transcriptionally, resulting in the compensatory upregulation of efflux gene expression^32,33^.

To test our model experimentally, we determined whether azoffluxin directly inhibited transporter function by monitoring accumulation within *C. auris* of the relatively promiscuous efflux pump substrate Nile red^34^. Flow cytometry revealed an 18.7-fold increase in relative cell-associated Nile red signal caused by treatment with azoffluxin (50 μM; Fig. 2d, e). To determine if Cdr1 and/or Cdr4-1 were relevant targets of azoffluxin, we utilized a *C. auris* strain in which *CDR1* had been deleted^35^, and also generated a *CDR4*-*1* deletion strain. If azoffluxin acts by inhibiting the activity of either transporter, then deletion of that transporter should reduce or eliminate the increase in Nile red accumulation caused by azoffluxin treatment. Although deletion of these efflux genes did not completely block the increase in Nile red accumulation caused by azoffluxin treatment, the magnitude of the increase was significantly diminished (*p* < 0.05) in the *cdr1Δ* strain (Figs. 2e, S2, and S3a), implicating Cdr1 as a likely target of azoffluxin in *C. auris*. It is possible that other *C. auris* transporters are also targets of azoffluxin, as Nile red signal was still enhanced upon azoffluxin treatment in the absence of *CDR1* or *CDR4-1*. However, Cdr4-1 is unlikely to be a relevant target given that loss of this transporter had no significant effect on the increase in Nile Red staining caused by azoffluxin.

Encouraged by the effects seen on Nile red as a model efflux substrate, we next assessed the functional relevance of Cdr1 for potentiation of fluconazole activity by azoffluxin. As would be expected if azoffluxin enhances fluconazole activity via inhibition of Cdr1, we found that deletion of *CDR1* abolished the ability of azoffluxin to potentiate the antifungal activity of fluconazole (Fig. 2f). In dose-response assays, deletion of *CDR1* reduced fluconazole MIC to that observed upon combination with azoffluxin in a wild-type background (Fig. 2f). In contrast, deletion of *CDR4*-1 did not alter fluconazole sensitivity nor the ability of azoffluxin to potentiate fluconazole (Fig. 2f). This finding fits with previous reports in *C. albicans* that implicate Cdr1 in azole efflux but not Cdr4-1, despite both being transcriptionally upregulated in response to fluconazole^36,37^.

**Fig. 3:**
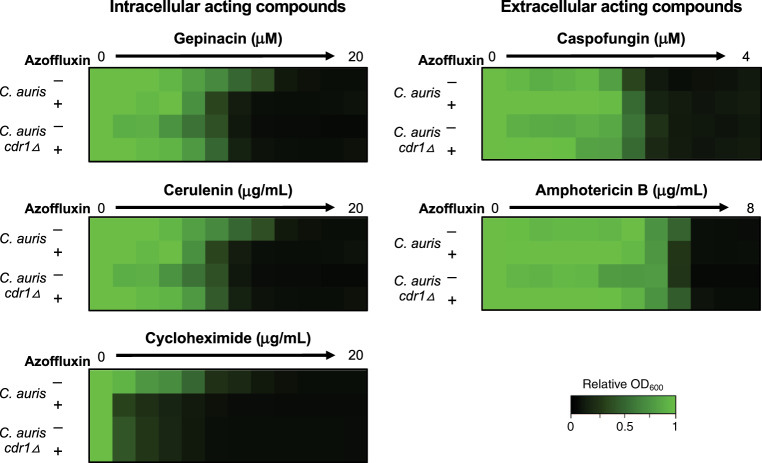
Azoffluxin potentiates intracellular acting compounds against *C. auris*, to a similar degree as deletion of *CDR1*. Dose-response assays were conducted with a *C. auris* Ci6684 parental strain in the absence and presence of 25 µM azoffluxin where indicated, as well as with a strain with the efflux pump gene *CDR1* deleted. Indicated compounds were titered in a 2-fold serial dilution. Growth was measured after 24 h in YPD as described in Fig. 1e (see color bar). Source data are provided as a Source Data file.

Given the extensive range of substrates that Cdr1 is reported to efflux, we investigated whether azoffluxin potentiated the effects of other intracellularly acting compounds to the same extent as deletion of *CDR1*. Consistent with our model for its mode of action, azoffluxin sensitized *C. auris* to the compounds gepinacin, cerulenin, and cycloheximide to the same extent as *CDR1* deletion, with no further sensitization to these compounds observed by azoffluxin in the *CDR1* null (Fig. 3). Although mechanistically diverse, these compounds all act intracellularly and are known to be Cdr1 efflux substrates^38,39^. In contrast, azoffluxin had no impact on sensitivity to the extracellularly acting compounds caspofungin and amphotericin B^10^ (Fig. 3). Considering all these findings, we conclude that azoffluxin enhances the antifungal activity of intracellularly acting compounds, such as fluconazole, by inhibiting drug transporters, most notably Cdr1, in *C. auris*.

### Azoffluxin is active against diverse *C. auris* strains

**Fig. 4:**
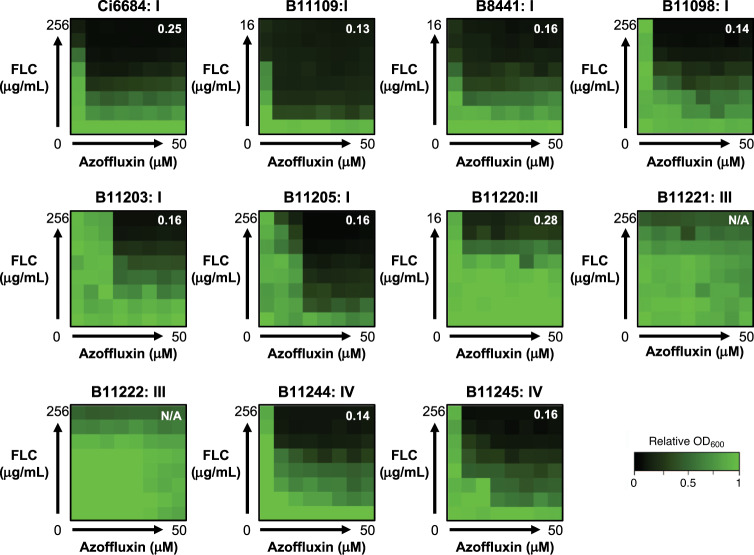
Synergistic activity of azoffluxin is clade specific. Checkerboard assays were performed fluconaozle (FLC) and azoffluxin as described in Fig. 1b with isolates from each major clade of *C. auris*. CDC identification number is followed by the clade number to which the isolate belongs. Relative growth was measured in YPD medium after 24 h using OD_600_ and normalized to a no-drug control well (see color bar). The FICI calculated for each checkerboard is shown in the top right of each plot, with values <0.5 indicating synergy, values >0.5 indicating no interaction, and N/A indicating an FICI that could not be calculated due to a lack of growth inhibition. Source data are provided as a Source Data file.

**Fig. 5:**
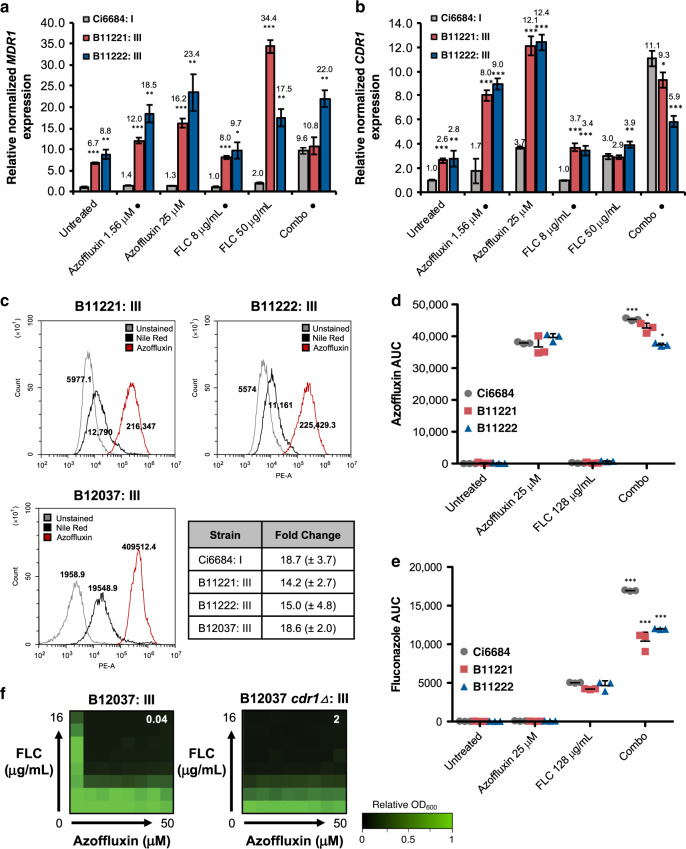
Azoffluxin does not potentiate fluconazole (FLC) against most clade III isolates despite intracellular FLC accumulation and increased *CDR1* expression. **a** Relative transcript levels of *MDR1* (B9J08_003981) and **b** relative transcript levels of *CDR1* (B9J08_000164) were measured in clade I isolate Ci6684 (gray) and clade III isolates B11221 (red) and B11222 (blue) (• indicates concentrations of FLC and azoffluxin used in combination (combo) treatment). Data are presented as mean ± SEM of technical triplicates. A two-sided unpaired Student’s *t* test was performed to evaluate significance of differences between Ci6684 and each clade III isolate *p-value < 0.05, **p-value < 0.01, and ***p-value < 0.001. **c** Flow cytometry was used to measure the Nile red accumulation in *C. auris* clade III strains as described in Fig. 2e. Values in histogram plots depict median fluorescence intensity (MFI) and table shows the mean fold-change in MFI ± SD for biological triplicates. **d** The relative intracellular azoffluxin abundance and **e** the relative intracellular FLC abundance was quantified by LC-MS as described in Fig. 2b in Ci6684 (gray), B11221 (red), and B11222 (blue). Data are presented as mean ± SD of technical triplicates. Significance of differences between azoffluxin and the combination treatment for each strain was determined by two-sided unpaired Student’s *t* test, *p-value < 0.05, and ***p-value < 0.001 comparing. **f** Checkerboard assay as described in Fig. 1b using parental clade III isolate B12037 and the strain with *CDR1* deleted, in YPD medium. Relative growth was measured after 24 h as described in Fig. 1b (see color bar). The FICI for each checkerboard is shown as described in Fig. 4. Source data are provided as a Source Data file.

Given the extensive genetic diversity identified amongst different clades of *C. auris*^3,7,18^, we investigated whether azoffluxin synergized with fluconazole against representative isolates from all four major clades. Intriguingly, when synergistic activity was assessed by checkerboard assay, azoffluxin potentiated fluconazole in multiple isolates from three of the four major clades. The clade III isolates from South Africa were the exception (Fig. 4). Clade III is generally distinguishable from the others by both a V125A and F126L substitution in Erg11^7^ and the absence of drug-resistance mutations in *TAC1B*, the transcriptional regulator of Cdr1 which are commonly found in clades I and IV^21^ (e.g. the A640V substitution in our screening strain VPCI 673/P/12). Examining whole genome sequences of 304 isolates representing each of the four major clades^7^, identified a unique non-synonymous substitution, N647T, in the transcription factor domain of Mrr1 (B9J08_004061) in 49 of 51 clade III isolates. In *C. albicans*, Mrr1 is a transcription factor that controls the expression of the major facilitator superfamily (MFS) transporter Mdr1, which is involved in fluconazole efflux^40,41^. Indeed, when expression of *MDR1* (B9J08_003981) was assessed in the clade III isolates B11221 and B11222, we observed a >6-fold increase in expression under nearly all conditions tested compared to the clade I screening strain Ci6684, suggesting that *MDR1* is constitutively upregulated in clade III isolates (Fig. 5a). In addition, despite the absence of known *TAC1* activating mutations in clade III isolates, we observed increased expression of *CDR1* in both clade III isolates relative to Ci6684 under all treatment conditions except for the drug combination (Figs. 5b and S3b). The lack of fluconazole potentiation by azoffluxin in clade III isolates coupled with the observation that efflux pump expression is high in these strains suggested an efflux-independent fluconazole-resistance mechanism in strains B11221 and B11222. To probe whether azoffluxin is able to inhibit drug efflux activity in clade III strains, cellular accumulation of Nile red, which is a substrate for both ABC and MFS efflux pumps^34^, was measured in the absence and presence of compound. Treatment with azoffluxin led to a 14.2-15-fold increase in Nile red accumulation in B11221 and B11222 (Fig. 5c), which was not significantly different in magnitude from the increase observed with the clade I screening isolate Ci6684 (Fig. 2e). Furthermore, LC-MS confirmed that azoffluxin accumulated intracellularly in B11221 and B11222 (Fig. 5d), and levels of intracellular fluconazole were significantly increased by combination treatment compared to fluconazole alone (Fig. 5e). These results suggest the lack of fluconazole potentiation by azoffluxin in clade III isolates is not due to an inability of the compound to inhibit efflux pumps, but rather that azole resistance in these isolates is not due solely to efflux.

To investigate whether the Erg11^V125A/F126L^ and/or Mrr1^N647T^ substitutions were likely responsible for resistance to the fluconazole-enhancing effects of azoffluxin in clade III isolates, we assessed the activity of azoffluxin against a clade III isolate (B12037) that does not contain the Erg11 substitutions or the Mrr1 substitution shared by most members of this clade^7^. While more sensitive to fluconazole than other clade members at baseline, this strain showed increased Nile red accumulation upon treatment with azoffluxin (Fig. 5c) and showed potent synergistic interaction between azoffluxin and fluconazole (Fig. 5f). Furthermore, when *CDR1* was deleted in this background, it abolished the synergistic activity (Fig. 5f), highlighting the importance of Cdr1 for azole tolerance of this clade III isolate. Together, our results suggest that substitutions in Erg11 and/or Mrr1 in B11222 and B11221 enable fluconazole resistance which is recalcitrant to the effects of azoffluxin.

### Azoffluxin displays activity against azole-resistant *C. albicans*

**Fig. 6:**
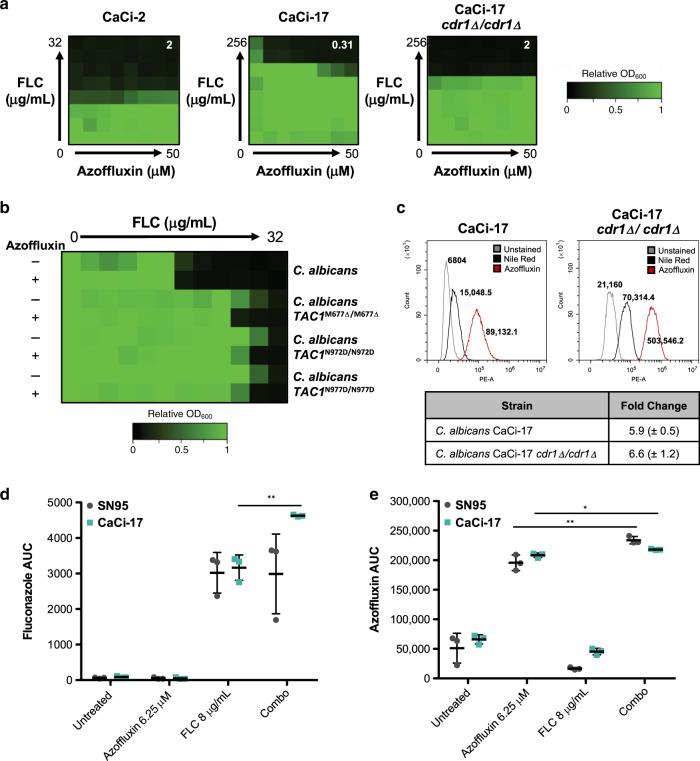
Azoffluxin enhances fluconazole (FLC) activity against azole-resistant *C. albicans* isolates. **a** Checkerboard assays as described in Fig. 1b were performed in YPD with isolates of *C. albicans*. Strains CaCi-2 and CaCi-17 represent early and late clinical isolates in which FLC resistance evolved over time. Growth was measured after 24 h using OD_600_ and normalized to a no-drug control well (see color bar). The FICI calculated for each checkerboard is shown in the top right of each plot, with values <0.5 indicating synergy and >0.5 indicating no interaction. **b** Dose-response assays were conducted in YPD medium with a *C. albicans* parental strain, and strains with gain of function mutations in *TAC1* as indicated. FLC was titered in a 2-fold dilution on the *x*-axis in the absence and presence of 50 µM azoffluxin. Growth was measured at 24 h using OD_600_ and normalized to a no-drug control well (see color bar). **c** Flow cytometry was used to measure relative Nile red accumulation in *C. albicans* strains as described in Fig. 2e. Values in histogram plots depict median fluorescence intensity (MFI) and table shows the mean fold-change in MFI ± SD for biological triplicates. **d** Relative intracellular levels of FLC and **e** relative intracellular azoffluxin were measured by LC-MS in *C. albicans* strains SN95 (gray) and CaCi-17 (blue) after treatment (combo treatment: 6.25 µM azoffluxin, 8 µg/mL FLC) for 1 h as described in Fig. 2b. Data are presented as mean ± SD of technical triplicates. Significance of differences was determined by a two-sided unpaired Student’s *t* test comparing fluconazole alone to the combination, *p-value = 0.013 and **p-value >  0.011. Source data are provided as a Source Data file.

Our studies so far supported a model in which azoffluxin inhibits Cdr1-dependent fluconazole resistance in *C. auris*. Notably, our initial findings suggested this compound combination was ineffective against *C. albicans, C. glabrata*, or *S. cerevisiae* (Fig. 1e), indicating either species-specific differences in the manner by which azoffluxin inhibits efflux pumps, or that efflux does not play a role in the azole sensitivity of the strains we tested. To learn whether azoffluxin had activity against strains of *C. albicans* in which fluconazole resistance is mediated through enhanced efflux, we assessed activity of the azoffluxin-fluconazole combination treatment against isolates from a patient who had received intermittent therapy with fluconazole over the course of 2 years^40,42,43^. We observed no potentiation in the early clinical isolate, CaCi-2 (Fig. 6a), which is reported to have no bona fide resistance mutations, consistent with our finding of no potentiation in an azole-sensitive laboratory strain, SN95 (Fig. 1e). Interestingly, azoffluxin did potentiate fluconazole against the late clinical isolate, CaCi-17, which possesses the substitutions A736V in Tac1 and G947S in Mrr1 that lead to upregulation of multiple efflux genes, in addition to gain-of-function mutations in the transcription factor *UPC2* that lead to overexpression of Erg11^R467K^^44,45^. The ability of azoffluxin to potentiate fluconazole in CaCi-17 was abolished upon deletion of *CDR1* (Fig. 6a), similar to what we observed in *C. auris* (Fig. 2f). Additionally, azoffluxin was able to mildly potentiate fluconazole in three laboratory-generated *C. albicans* strains with verified gain-of-function mutations in *TAC1*^46^, but not the sensitive parental strain (Fig. 6b).

To confirm that azoffluxin inhibited efflux in *C. albicans*, we assessed Nile red accumulation in CaCi-17 by flow cytometry. We observed a similar increase in Nile red accumulation upon azoffluxin treatment in both the parental CaCi-17 and the CaCi-17 *cdr1Δ/cdr1Δ* strain (Fig. 6c). This suggests that while azoffluxin is able to inhibit efflux pumps in *C. albicans*, Cdr1 is either not the major Nile red transporter or other transporters are able to compensate upon its deletion. Finally, to confirm that combination treatment was blocking efflux and resulting in increased fluconazole accumulation, we used LC-MS to measure intracellular compound concentrations in SN95, which was recalcitrant to the potentiation effects of azoffluxin (Fig. 1e), and CaCi-17. Comparing combination treatment to that with each compound alone, we only detected a significant increase in fluconazole in CaCi-17 (Fig. 6d), while in both strains there was a significant increase in azoffluxin (Fig. 6e). These data indicate that azoffluxin blocks fluconazole efflux in a resistant clinical isolate of *C. albicans*, increasing its sensitivity to fluconazole, establishing bioactivity for azoffluxin beyond *C. auris*.

### Combination treatment reduces fungal burden in vivo

**Fig. 7:**
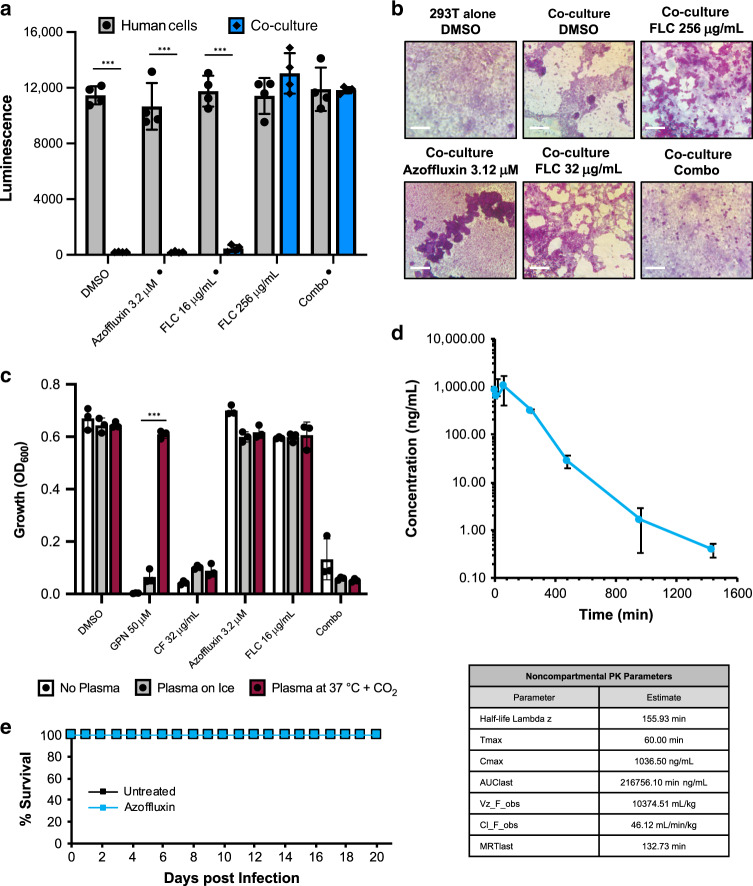
Preliminary characterization of the in vivo potential of azoffluxin. **a** Human cells (293T) expressing luciferase were grown in DMEM medium overnight. 24 h later the indicated concentrations of compounds (• indicates concentrations of fluconazole (FLC) and azoffluxin used in combination (combo) treatment) were added to cells alone (gray) or those infection with *C. auris* (blue). Co-cultures were incubated for 48 h at 37 °C followed by measurement of luminescence. Data are presented as mean ± SD of quadruplicate wells. Significance of differences between 293T cells alone versus co-cultures was determined by two-sided unpaired Student’s *t* test, (***p-value < 0.001 **b** Periodic-Acid Schiff (PAS) staining was used to visualize cells in co-culture. Light purple staining identifies 293 T cells and the bright pink signal indicates *C. auris*. Scale bar represents 50 µm. **c** Plasma stability of azoffluxin and relevant control compounds, gepinacin (GPN) and caspofungin (CF). Samples were incubated in 100% mouse plasma at either at 37 °C with 5.5% CO_2_ (maroon) or on ice (gray), or in the absence of serum in YPD (black). The drug-plasma mixtures were diluted 1:10 into *C. auris* Ci6684-inoculated YPD medium. Relative growth was measured after 48 h at 30 °C by OD_600_. Data are presented as mean ± SD between technical triplicates, two-sided unpaired Student’s *t* test was used to determine significance of difference between 37 °C with 5.5% CO_2_ condition compared to ice condition for each treatment, ***p-value < 0.001. **d** Plasma concentrations of azoffluxin in mice following intraperitoneal (IP) bolus administration of compound (10 mg/kg). Azoffluxin was quantitated in mouse blood (*n* = 3) by LC-MS/MS. Data are presented as mean ± SD of three mice. Pharmacokinetic properties shown in the table below were evaluated using Analyst software (AB Sciex.) and the noncompartmental analysis tool in WinNonlin (Certara, Corp.). **e** Tolerability of azoffluxin in mice was evaluated by treating neutropenic ICR (CD-1) mice with azoffluxin 10 mg/kg IP twice daily for 4 days and monitoring the health and survival of treated (blue) and untreated (black) mice (*n* = 5) for 21 days. Source data are provided as a Source Data file.

Encouraged by the intriguing mode of action we had uncovered, we next assessed the therapeutic potential of combining azoffluxin with fluconazole. We first examined the ability of this combination to rescue human kidney-derived (293T) cells when infected with fungus^47^. 293T cells constitutively expressing firefly luciferase as a reporter were either grown alone or in co-culture with *C. auris* Ci6684 under various treatment conditions. Luminescence was used as an indicator of relative viable human cell number. In the cases of solvent control, azoffluxin alone, or low fluconazole, *C. auris* growth was unhindered, which resulted in near complete human cell loss and an absence of luminescent signal (Fig. 7a). However, with combination treatment (azoffluxin and low fluconazole) we saw dramatic rescue of the human cells, comparable to that achieved with a 16-fold higher concentration of fluconazole alone (Fig. 7a). Notably, for 293T cells grown in the absence of *C. auris* we saw no significant change in luciferase signal under any treatment condition, indicating minimal cytotoxicity in vitro (Fig. 7a).

The same experimental design was performed in 24-well plate format followed by Periodic-acid Schiff (PAS) staining of polysaccharides to visualize effects of the various treatments on both the fungal and human elements within the co-cultures. Results supported findings obtained using the quantitative assay. We observed extensive damage to the human cell monolayer (stained pale purple) and sloughing in conjunction with the presence of abundant *C. auris* (stained pink) in wells exposed to azoffluxin alone or low fluconazole alone, comparable to the untreated co-culture. With combination treatment, we observed an intact human cell monolayer which was similar to the no fungus control, and scant fungal burden. In the high fluconazole condition, some disruption of the human cell monolayer was evident and fungal cells were readily apparent, suggesting that high fluconazole was effective at reducing fungal toxicity to the monolayer, but less effective than our combination treatment in arresting fungal proliferation (Fig. 7b).

Justified by promising results in culture, the therapeutic potential of azoffluxin was investigated in mice. First, we assessed the stability of azoffluxin in mouse plasma by bio-assay. Incubation of the compound in 100% mouse plasma for 1 h caused no decrease in azoffluxin potentiating activity. Technical controls consisted of the experimental antifungal gepinacin, which is readily inactivated by plasma^48^, and caspofungin, a stable clinical antifungal (Fig. 7c). Next, a single dose pharmacokinetic study was performed in mice to inform design of appropriate regimens to assess tolerability and efficacy. Following a 10-mg/kg bolus dose, the peak plasma concentrations of azoffluxin achieved were well above those required for azole-potentiating activity in vitro (>1 µg/mL) and a half-life of ~ 2.6 h was defined (Fig. 7d). These findings encouraged us to proceed with a repeated-dose tolerability study which confirmed the absence of any physical signs of acute systemic toxicity after 4 days of treatment and 100% survival after 21 days (Fig. 7e).

**Fig. 8:**
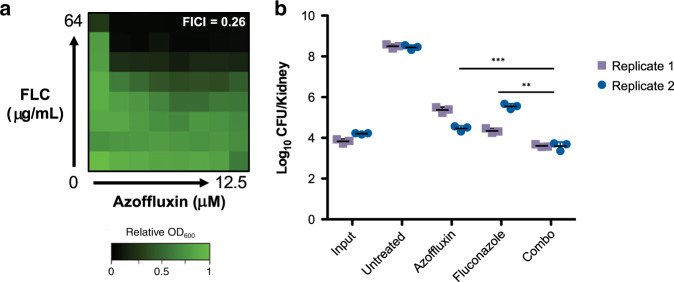
Azoffluxin increases the antifungal activity of fluconazole (FLC) in mice. **a** Checkerboard assays were performed as described in Fig. 1b with *C. auris* clade IV isolate B11801. Relative growth was measured after 24 h using OD_600_ and normalized to no-drug control wells (see color bar). The FICI is shown in the top right of each plot, with values <0.5 indicating synergy. **b** Kidney fungal burden (CFU) in mice from each treatment group that had been infected with *C. auris* B11801. Input is the CFU recovered in an aliquot of the fungal suspension used to inoculate mice. All other values are the CFU recovered from kidney homogenates after 4 days of treatment. Fluconazole was administered at 32 mg/kg intraperitoneally twice daily and azoffluxin at 10 mg/kg subcutaneously four-times daily. Data are presented as mean ± SD of *n* = 3 mice per treatment group. Experiment was performed in two independent replicates (purple and blue). The significance of differences between combination treatment and treatment with each compound alone was determined for each replicate by two-sided unpaired Student’s *t* test, **p-value < 0.01 and ***p-value < 0.001. Source data are provided as a Source Data file.

Finally, we evaluated the efficacy of azoffluxin in a well-characterized mouse model of systemic *C. auris* infection^49^. Immunocompromised mice were infected intravenously with the azole-resistant clade IV isolate B11801, which we had confirmed as susceptible to the fluconazole-potentiating effects of azoffluxin (Fig. 8a). After four days of well-tolerated treatment, fluconazole alone reduced kidney colony forming units (CFU) compared to untreated mice, however, the addition of azoffluxin significantly enhanced this activity (*p*-value < 0.001; Fig. 8b) compared to either treatment alone. Unexpectedly, azoffluxin alone reduced fungal burden by ~3-log_10_ CFU despite having shown no effect on *C. auris* growth in vitro (Fig. 8). This surprising result suggests that disruption of Cdr1 function by azoffluxin impairs *C. auris* virulence in immunocompromised mice, as has been previously demonstrated using genetic approaches in isolates of *C. glabrata*^50^. Overall, azoffluxin displays antifungal activity in vivo, further validating the concept of targeting resistance mechanisms in combination with current antifungals to combat *C. auris* infections.

## Discussion

In this study, we leveraged a diversity-oriented chemical library to discover CMLD012336 (azoffluxin) as a new compound that enhances the susceptibility of resistant fungal pathogens to diverse intracellularly acting antifungals. This compound inhibits the activity of multidrug efflux transporters, most notably the ABC transporter Cdr1. Enhanced multidrug efflux is a frequently encountered and problematic mechanism of antimicrobial resistance^36,39,51–53^. With the escalating problem of antifungal resistance to public health, the ability of azoffluxin to inhibit drug efflux in a non-toxic manner could have potential therapeutic implications.

The 3,3-diarylated oxindole we named azoffluxin was identified as an unexpected side product in a Lewis-acid mediated Friedel-Crafts/Prins reaction process intended to generate spirocyclic oxindoles^54^. 3,3-diarylated oxindoles are a subset of the medicinally “privileged” 3,3-disubstituted oxindole class, which have a rich history of reported biological activities. For example, the diphenolic oxindole oxyphenisatin and other 3,3-diarylated oxindoles have been widely reported to inhibit the growth of diverse cancer cell lines, in many cases the activity being ascribed to inhibition of eIF2α-mediated translation initiation^55,56^. Another 3,3-diarylated oxindole BHPI, has been reported to act as a non-classical agonist of human estrogen receptor α and was recently shown to deplete intracellular ATP in estrogen receptor-positive cancer cells, thereby disrupting ATP-dependent ABC transporter-mediated drug efflux^57^. Whether BHPI might directly inhibit human or fungal ABC transporters is unknown. Other 3,3-bisaryl oxindoles have been reported as mineralocorticoid receptor antagonists^58^, as well as antioxidants^59^. Although azoffluxin falls into the general class of 3,3-disubstituted oxindoles, its specific substituents impart structurally distinct features to the compound. In addition to differences at the level of chemical structure, divergence in the processes reported to be impacted by other class members between mammalian and fungal cells, and azoffluxin’s lack of cytotoxicity to human cells and good tolerability in mice make it unlikely that azoffluxin operates through the mechanisms previously described for other 3,3-disubstituted oxindoles.

Using azoffluxin as a chemical probe, we found that *C. auris* strains belonging to clades I, II, and IV were sensitized to fluconazole, implicating efflux as a major factor contributing to their high azole-resistance. Indeed, inhibition of efflux in these strains markedly reduced their fluconazole resistance, despite the presence of Erg11 alterations, most notably Erg11^Y132F^ and Erg11^K143R^ that have been reported to cause a >4-fold increase in azole MIC^16^. Furthermore, azoffluxin reduced azole resistance of a *C. albicans* strain harboring numerous resistance-conferring mutations in genes such as *ERG11*, *TAC1*, *MDR1*, and *UPC2*^44,45^. Deletion of *CDR1* in specific strains of *C. auris* and *C. albicans* abolished azoffluxin-mediated potentiation. In contrast, we did not observe azoffluxin-mediated fluconazole potentiation in the more divergent fungal species *C. glabrata* and *S. cerevisiae*. One explanation would be that Pdr5, the homologous efflux pump in these species, is not inhibited by azoffluxin, which is plausible given the relatively low 56% sequence identity between Cdr1 and Pdr5^60^. Alternatively, efflux may not be a driving factor in determining the fluconazole sensitivity of the strains used as representatives of these species.

Within *C. auris*, we found that isolates from clade III were distinct in their lack of susceptibility to the fluconazole-sensitizing effect of azoffluxin. Clade III isolates often express an Erg11 variant with V125A/F126L substitutions and harbor a unique substitution in Mrr1 (N647T), which likely leads to upregulation of the MFS pump Mdr1^7^. Unlike clade I and IV, this clade does not possess candidate drug-resistance substitutions in Tac1b^21^. While we confirmed upregulation of *MDR1* and *CDR1* in clade III isolates compared to clade I, azoffluxin treatment of these strains still increased accumulation of fluconazole, and Nile red which is a substrate of both Mdr1 and Cdr1. The mechanism(s) underlying this observation is unclear but include the possibility that azoffluxin inhibits Mdr1 as well as Cdr1. Alternatively, inhibition of Cdr1 in these strains might be sufficient to increase compound accumulation despite Mdr1 upregulation, or azoffluxin is able to inhibit the function of yet other efflux pumps for which these compounds are also a substrate^34^. Irrespective of their mechanism, our findings and the observation that azoffluxin synergized with fluconazole against an atypical clade III isolate that did not carry the Erg11^V125A/F126L^ or Mrr1^N647T^ substitutions suggest that one or more of these resistance-conferring mutations is responsible for the inability of azoffluxin to potentiate fluconazole activity against most clade III isolates. In light of a previous report that Mdr1 does not play a role in fluconazole resistance of clade I isolates^20^ and our finding that azoffluxin enhanced fluconazole accumulation in clade III isolates, it is likely that the impact of Mdr1 upregulation on azole resistance in clade III strains is negligible. Rather, the specific Erg11^V125A/F126L^ mutant target protein in most clade III strains is likely the dominant determinant of their fluconazole resistance and would explain the inability of an efflux pump inhibitor such as azoffluxin to restore azole sensitivity.

Efflux is regulated by complex and highly interconnected genetic circuitry. Recent analyses of *S. cerevisiae* genetic interaction networks show that perturbation through deletion of specific ABC transporter genes can paradoxically lead to an increase in azole resistance^61^. This response was mediated, at least in part by compensatory upregulation of *PDR5* expression, as deletion of *PDR5* restored fluconazole sensitivity^61^. We find that expression of efflux genes such as *CDR1* and *CDR4-1* were upregulated in *C. auris* in response to azoffluxin, while genes such as *SNQ2*-2 were downregulated. This pattern highlights the complex connectivity of the efflux network as well as the potential contribution of general stress responses to efflux pump expression. Regardless, this upregulation did not preclude the ability to sensitize cells to fluconazole. The robust activity of azoffluxin despite the compensatory upregulation of efflux genes reflects the strong dependence of high level fluconazole-resistance on Cdr1^20,61,62^. Consistent with this dependence, the highly resistant *C. albicans* clinical isolate CaCi-17 was susceptible to azoffluxin-fluconazole combination treatment. In contrast, azoffluxin had no impact on azole sensitivity of the earlier clinical isolate CaCi-2, which possesses no bona fide mutations and is far more susceptible to fluconazole. Such insights underscore the value of azoffluxin as a chemical probe to discern the relative role of efflux in mediating antifungal drug resistance across diverse fungal pathogens.

From a therapeutic perspective, utilizing a chemical combination in which one compound targets an essential process and the other disables a major resistance mechanism provides an attractive strategy that has been explored for both antimicrobial and cancer treatment^39,52,63–66^. In the case of efflux inhibitors, not only does this strategy enhance the efficacy of the other compound, but if applied early in the course of intervention, it can also reduce the rate at which resistance emerges^39,67^. Despite the conceptual appeal, no efflux inhibitor combination therapies have proven effective in patients^52^. This failure in clinical translation has largely been due to host toxicity, off target effects, and/or the poor pharmacokinetics that have plagued current efflux inhibitors such as verapamil, cyclosporin A, and valspodar^39,66^. In this study, we found azoffluxin to be very well tolerated both in culture and in mice. As well, azoffluxin significantly enhanced fluconazole activity in an immunocompromised mouse model of invasive *C. auris* infection, and reduced fungal burden by ~1000-fold even as a single agent, consistent with the role of Cdr1 in virulence of *C. glabrata*^50^.

An additional distinct challenge to the efficacy of efflux inhibitors is the emergence of target-based and other resistance mechanisms that can render efflux a less important factor contributing to the overall resistance level of a fungal pathogen^52^. Encouragingly, azoffluxin remains able to sensitize azole-resistant *Candida* strains despite the presence of target-based resistance mechanisms. While overall results are encouraging, they are clearly discovery-phase in nature and will require further development for translation to clinical application. As the number of drug-resistant infections continues to rise, there remains a need to understand the relative contribution of different resistance mechanisms to the diminishing efficacy of our limited antifungal armamentarium and to design new resistance-evasive treatment strategies.

## Methods

### Strain construction

All strains, plasmids, and oligonucleotides used in this study are listed in Supplementary Information.

### CauLC6410: *C. auris* Ci6684 *cdr4-1△*::NAT

The *C. auris* strain with *CDR4*-1 (B9J08_000479) deleted was constructed using homologous recombination and an electroporation transformation approach^35^. Approximately 1 kb of sequence homology upstream of *CDR4*-1 was amplified using primers oLC8164/oLC8165 and ~1 kb of homology downstream of *CDR4*-1 was amplified with oLC8166/oLC8167. The interior primer of each set contained 40 bp homology to a nourseothricin (NAT) resistance marker from pLC1049, which was amplified with primers oLC6296/oLC6304. Using fusion PCR with nested primers oLC8168/oLC8169, the NAT cassette and *CDR4*-1 homology regions were combined into a single DNA fragment. This PCR product was ethanol precipitated. *C. auris* CaLC5083 cells were prepared by growing in 50 mL YPD medium to an OD_600_ of 1.6–2.2. Cells were pelleted for 5 min at 3000×*g*, then subcultured for 1 h in 10 mL 10 mM Tris-HCl (Bioshop), and 1 mM EDTA (Bioshop) in ddH_2_O (1x TE buffer) and 0.1 M Lithium Acetate (Sigma). In all, 250 µL of 1 M DTT was added to the culture for 30 min. Cells were washed twice in cold ddH_2_O followed by a wash in cold 1 M sorbitol (Bioshop). In all, 3 μg of DNA was electroporated into 40 µL CaLC5083 cell suspension in a 2-mm electroporation cuvette (VWR) with the following settings on a BTX ECM830 electroporator: 1.8 kV, 200 Ω, 25 µF and outgrown in YPD medium for 4 h at 30 °C. Transformants were plated on YPD plates containing 150 μg/mL NAT. Colonies were patched and genotyped for integration of the deletion construct (oLC8164/oLC6308 and oLC274/oLC8167) and for the absence of the wild-type allele (oLC8052/oLC8053).

### CaLC5447: *C. albicans* CaCi-17 *cdr1△/cdr1△*

Both alleles of *CDR1* (C3_05220W) were deleted by a transient CRISPR method^68^. The guide construct was made of two components from pLC1081: the *SNR52* promoter amplified with the universal primer oLC6929 and guide specific primer oLC6966, and the guide scaffold and terminator amplified with the guide specific primer oLC6967 and universal primer oLC6927. The fusion construct was PCR amplified with the universal nested primers oLC6928/olC6929^69^. Repair template was digested from pLC1083 by ApaI (NEB) and SacI (NEB). Gene deletion was verified by the absence of the *CDR1* specific amplicon with oLC6968/oCL6969^69^.

### CaLC5589: *C. albicans* SN152 *TAC1/TAC1*

Stepwise insertion of wild-type *TAC1* back into the *tac1△/tac1△* deletion mutant CaLC4255^70^ to match gain-of-function strains below. oLC1096 was digested by BamHI (NEB) and SacII (NEB) and used to transform CaLC4255. NAT resistant transformants was further triaged by a histidine auxotrophy. Re-introduction of the *TAC1* ORF was verified by the presence of the oLC7041 and oLC7042 amplicon. Subsequently, pLC1092 was digested by BamHI and SacII and used in a second transformation. Transformants prototrophic for histidine were further selected for NAT resistance.

### CaLC5591: *C. albicans* SN152 Tac1^M677△^/Tac1^M677△^

Stepwise insertion of a *TAC1*^*A2029-G2031△*^ (Tac1^M677*△*^ allele) into the *tac1△/ tac1△* deletion mutant CaLC4255. oLC1097 was digested by BamHI and SacII and used to transform CaLC4255. NAT resistant transformants was further triaged by a histidine auxotrophy. Re-introduction of the *TAC1*^*A2029-G2031△*^ ORF was verified by the presence of the oLC7041 and oLC7042 amplicon. Subsequently, pLC1093 was digested by BamHI and SacII and used in a second transformation. Transformants prototrophic for histidine were further selected for NAT resistance.

### CaLC5593: *C. albicans* SN152 Tac1 ^N972D^/Tac1 ^N972D^

Stepwise insertion of a *TAC1*^A2914G^ (Tac1^N972D^ allele) into the *tac1△/ tac1△* deletion mutant CaLC4255. oLC1098 was digested by BamHI and SacII and used to transform CaLC4255. NAT resistant transformants was further triaged by a histidine auxotrophy. Re-introduction of the *TAC1*^A2914G^ ORF was verified by the presence of the oLC7041 and oLC7042 amplicon. Subsequently, pLC1094 was digested by BamHI and SacII and used in a second transformation. Transformants prototrophic for histidine were further selected for NAT resistance.

### CaLC5595: *C. albicans* SN152 Tac1^N977D^/Tac1^N977D^

Stepwise insertion of a *TAC1*^A2929G^ (Tac1^N977D^ allele) into the *tac1△/ tac1△* deletion mutant CaLC4255. oLC1099 was digested by BamHI and SacII and used to transform CaLC4255. NAT resistant transformants was further triaged by a histidine auxotrophy. Re-introduction of the *TAC1*^A2929G^ ORF was verified by the presence of the oLC7041 and oLC7042 amplicon. Subsequently, pLC1095 was digested by BamHI and SacII and used in a second transformation.

Transformants prototrophic for histidine were further selected for NAT resistance.

### CauLC6750: *C. auris* B12037 *cdr1△*::NAT

To delete *CDR1* (B9J08_000164) in *C. auris* B12037 a homologous recombination and electroporation transformation approach was used^35^. Approximately 1 kb of sequence homology upstream of *CDR1* was amplified using primers oLC6020/oLC6305 and ~1 kb of homology downstream was amplified with oLC6306/oLC6025. The interior primer of each set contained 40 bp homology to a nourseothricin (NAT) resistance marker from pLC1049, which was amplified with primers oLC6296/oLC6304. Using fusion PCR with nested primers oLC6024/oLC6307, the NAT cassette and *CDR1* homology regions were combined into a single DNA fragment. This PCR product was ethanol precipitated, 5 μg of DNA was electroporated into CauLC6554, and transformants were plated on YPD plates containing 150 μg/mL NAT. Colonies were patched and genotyped for integration of the deletion construct (oLC6221/oLC6308 and oLC274/oLC6023) and for the absence of the wild-type allele (oLC6231/oLC6169).

### Statistics and reproducibility

All data presented in this study are derived from two biological experiments in which both results agreed, and data shown in the figures are from technical replicates from a single biological replicate, representative of both, unless otherwise stated. All statistical analysis was performed by a two-sided Student’s *t* test in Microsoft Excel (version 16.41) unless otherwise stated.

### Culture conditions

All fungal strains were stored in 25% glycerol in YPD medium (YPD: 1% yeast extract, 2% peptone, and 2% D-glucose) and maintained at −80 °C. Strains were grown in either YPD or RPMI medium (10.4 g/L RPMI-1640, 3.5% MOPS, 2% D-glucose, supplemented with an additional 5 mg/mL histidine as required, pH 7). The mammalian cell line of human embryonic kidney 293T cells was stored in glycerol and cultured in DMEM medium (Sigma) with 10% fetal bovine serum (FBS; Gibco).

### BU-CMD library screen

A set of 2,454 compounds from the Boston University Center for Molecular Discovery (BU-CMD) library were used to identify compounds that enhance fluconazole activity against *C. auris*. All compounds were dissolved in DMSO (dimethyl sulfoxide; Sigma) at 5 mM. RPMI medium alone or containing 128 μg/mL fluconazole (MIC > 256 μg/mL; Sequoia Research Products) was inoculated with ~1 × 10^3^ cells/mL of *C. auris* (VPCI 673/P/12) from a saturated overnight culture. Both types of media were dispensed at 100 μL per well into 96-well, flat bottom, microtiter plates (Sarstedt). In total, 1 μL of DMSO-solubilized compound from the library was added into each well to a final concentration of 50 μM. Cells were incubated for 48 h at 30 °C and OD_600_ was read (Molecular Devices SpectraMax Plus 384). After the initial screen, all secondary chemical susceptibility assays were performed on fresh sample aliquots that were first assessed for purity by UPLC-MS-ELSD analysis.

### Chemical susceptibility assays

Compound potency was assessed alone by dose-response assays or in combination with another compound by dose-response matrixes in 96-well plates, or 384-well, flat bottom, microtiter plates (Corning)^71^. Plates were incubated at 30 °C for the indicated time period. Growth was quantified by measuring OD_600_ and corrected for medium background. All strains were assessed in biological duplicate experiments with technical duplicates. Growth was normalized to untreated controls and plotted as a heat map using Java TreeView (version 1.1.6r4). For dose-response matrixes fractional inhibitory concentration index at 90% growth inhibition (FICI_90_) was calculated using the formula: $\left( {\frac{{{\mathrm{MIC}}_{{\mathrm{Drug}}\,{\mathrm{A}}\,{\mathrm{Combo}}}}}{{{\mathrm{MIC}}_{{\mathrm{Drug}}\,{\mathrm{A}}\,{\mathrm{Alone}}}}} + \frac{{{\mathrm{MIC}}_{{\mathrm{Drug}}\,{\mathrm{B}}\,{\mathrm{Combo}}}}}{{{\mathrm{MIC}}_{{\mathrm{Drug}}\,{\mathrm{B}}\,{\mathrm{Alone}}}}}} \right)$^30^. The fluconazole Etest susceptibility assay was performed by plating 200 μL of 5 × 10^6^ cells/mL on YPD agar (1%) plates with either 50 μM azoffluxin or containing DMSO^35^. Etest strips (bioMérieux) were placed on top after drying and plates were incubated for 24 h at 30 °C and imaged. Etest susceptibility assays were performed in biological duplicate.

BU-CMD hit compounds were newly supplied and dissolved in DMSO. Gepinacin (Toronto Research Chemicals), cerulenin (Cayman Chemical Company), cycloheximide (BioShop), caspofungin (generously provided by Merck), and amphotericin B (Sigma) were dissolved in DMSO. Fluconazole was dissolved in sterile ddH_2_O or DMSO.

### Chemical synthesis

#### **General methods**

^1^H NMR spectra were recorded at 400 or 500 MHz at ambient temperature unless otherwise stated. ^13^C NMR spectra were recorded at 100 or 125 MHz at ambient temperature unless otherwise stated. Chemical shifts are reported in parts per million. Data for ^1^H NMR are reported as follows: chemical shift, multiplicity (app = apparent, br = broad, s = singlet, d = doublet, t = triplet, q = quartet, sxt = sextet, m = multiplet, ovrlp = overlap), coupling constants, and integration. All ^13^C NMR spectra were recorded with complete proton decoupling. Analytical thin layer chromatography was performed using 0.25 mm silica gel 60-F plates. Flash chromatography was performed using 200–400 mesh silica gel (Sorbent Technologies, Inc.) or prepack column (SI-HC, puriFlash) by Interchim. Isolated yields refer to chromatographically and spectroscopically pure compounds, unless otherwise stated. Analytical LC-MS experiments were performed using a Waters Acquity UPLC (ultra-performance liquid chromatography) with a binary solvent manager, SQ mass spectrometer, Waters 2996 PDA (photodiode array) detector and evaporative light scattering detector (ELSD).

All compounds tested in biological assays were determined to be >95% pure by UPLC-MS-ELSD analysis. For validation, the screening hit CMLD012336 was resynthesized via Lewis-acid mediated condensation of 6-fluoro-3,3-dimethoxyindolin-2-one and (*R*)-1-(benzo[*d*][1,3]dioxol-5-yloxy)-4-methylpent-4-en-2-ol.

#### **6-Fluoro-3,3-dimethoxyindolin-2-one**

To a flame-dried 100 mL round bottomed flask equipped with a reflux condenser under an atmosphere of N_2_ was added 6-fluoroisatin (1.0 g, 6.06 mmol), trimethylorthoformate (729 µL, 6.66 mmol), and methanol (30 mL). *p*-Toluenesulfonic acid monohydrate (172.8 mg, 0.908 mmol) was added and the reaction was heated to reflux for 5.5 h. After cooling to ambient temperature, the reaction was diluted with diethyl ether and neutralized with a saturated solution of sodium bicarbonate. The organic layer was separated, and the aqueous layer was extracted twice with diethyl ether. The combined organics were dried over sodium sulfate, filtered, and concentrated under reduced pressure. The residue was taken up in diethyl ether, filtered over a pad of celite/Na_2_SO_4_, and concentrated to give a yellow solid. The crude yellow solid was purified flash column chromatography (SiO_2_, gradient elution 1 → 7% methanol/dichloromethane, Interchim PuriFlash 450) to give product 6-fluoro-3,3-dimethoxyindolin-2-one (1.06 g; 82.8% yield). LCMS m/z [M-OMe]^+^ 180. ^1^H NMR (400 MHz, CD_3_COCD_3_) δ 9.57 (br s, 1H), 7.44 (dd, *J* = 8.21, 5.47, 1H), 6.79 (m, 1H), 6.72 (dd, *J* = 8.99, 2.34, 1H), 3.48 (s, 6H). ^13^C NMR (126 MHz, CD_3_COCD_3_) δ 173.1, 166.4, 164.5, 145.0, 144.9, 128.3, 109.3, 109.1, 100.1, 99.9, 51.2. HRMS [M+Na]^+^ calcd. for C_10_H_10_FNO_3_Na 234.0545, found 234.0542.

#### **(*R*)-1-(Benzo[*d*][1,3]dioxol-5-yloxy)-4-methylpent-4-en-2-ol**

In a flame-dried 50 mL round bottom flask under an atmosphere N_2_ was stirred a suspension of copper iodide (65.4 mg, 0.21 mmol) in THF (2 mL) cooled to −40 °C using an acetonitrile/CO_2_ bath. To this suspension was added isopropenylmagnesium bromide (0.5 M in THF, 6.18 mL). The reaction was stirred at −40 °C for 35 min. Next, a solution of 5-[[(2 *R*)-oxiran-2-yl]methoxy]-1,3-benzodioxole (400 mg, 2.06 mmol) in THF was added dropwise. The reaction was stirred at −40 °C for 150 min. The brown colored mixture was quenched at −40 °C by dropwise addition of saturated aqueous ammonium chloride (0.4 mL). The product mixture was then filtered over a pad of Celite/SiO_2_/Na_2_SO_4_. This pad was eluted with 60% ethyl acetate in hexanes (100 mL) and ethyl acetate (60 mL) to afford the desired product (493 mg) as a colorless oil in quantitative yield. ^1^H NMR (400 MHz, CDCl_3_) δ 6.71 (d, *J* = 8.6 Hz, 1H), 6.53 (d, *J* = 2.7, 1H), 6.35 (dd, *J* = 8.4, 2.4, 1H), 5.93 (s, 2H), 4.91 (s, 1H), 4.85 (s, 1H), 4.14 (m, 1H), 3.92 (dd, *J* = 9.3, 3.7, 1H), 3.83 (dd, *J* = 9.3, 7.1, 1H), 2.32 (d, *J* = 6.6, 2H), 1.81 (s, 3H). ^13^C NMR (126 MHz, CDCl_3_) δ 154.1, 148.3, 141.7, 113.7, 107.9, 105.8, 101.2, 98.2, 72.8, 67.8, 41.9, 22.5. HRMS [M+Na]^+^ calcd. for C_13_H_16_O_4_Na 259.0946, found 259.0944.

#### **(6-Fluoro-3,3-*bis*(6-(((*R*)-2-hydroxy-4-methylpent-4-en-1-yl)oxy)benzo[*d*][1,3]dioxol-5-yl)indolin-2-one (azoffluxin; CMLD012336)**

In a two dram vial under N_2_ was stirred 6-fluoro-3,3-dimethoxy-indolin-2-one (70 mg, 0.33 mmol) and (*R*)-1-(benzo[*d*][1,3]dioxol-5-yloxy)-4-methylpent-4-en-2-ol (170 mg, 0.72 mmol) in dichloromethane (4.2 mL). To this reaction was added magnesium sulfate (325 mg, 2.70 mmol). The reaction was cooled to 0 °C in an ice bath and scandium(III) triflate (400 mg, 0.81 mmol) was added. The reaction was allowed to slowly warm to room temperature. After stirring at room temperature overnight, the reaction was filtered through a pad of Celite eluting with dichloromethane. After concentration in vacuo, the crude residue was purified by flash column chromatography (SiO_2_, gradient elution 15–45% acetone in hexanes, Interchim PuriFlash 450) to afford azoffluxin (84 mg, 40.9% yield). LCMS *m*/*z* [M+H]+ 620. At ambient temperature, azoffluxin exhibits multiple sets of broadened ^1^H and ^13^C NMR peaks due to rotamers by restricted rotation of the biaryl system. NMR peaks were found to coalesce upon heating to 150 °C. NMR chemical shifts at both temperatures are reported.

^1^H NMR (DMSO-d_6_, 400 MHz, 25 °C) δ 10.60 (br s, 1 H), 7.35-7.17 (m, 1H), 6.77-6.72 (m, 2H), 6.67-6.55 (m, 2H), 6.48 (br. s, 0.5 H), 6.14 (br. d, *J* = 12.5 Hz, 1H), 5.95-5.90 (m, 2H), 5.88-5.84 (m, 2H), 4.67-4.63 (m, 2H), 4.57-4.46 (m, 2H), 3.78-3.38 (m, 5H), 3.30-3.33 (m, 1H), 1.95-1.72 (m, 4H), 1.65-1.55 (m, 6H); ^13^C NMR (DMSO-d_6_, 100 MHz, 25 °C) δ 180.0, 179.8, 163.5, 163.3, 161.0, 160.9, 153.0, 152.5, 151.9, 151.6, 147.7, 147.5, 147.23, 147.17, 143.2, 143.0, 142.95, 141.2, 141.0, 140.8, 130.8, 130.3, 127.2, 127.1, 126.8, 126.6, 121.1, 120.7, 112.6, 112.3, 109.2, 108.25, 108.2, 108.0, 107.8, 107.6, 101.7, 101.6, 97.9, 97.6, 97.2, 97.0, 96.95, 96.8, 74.0, 73.5, 73.1, 72.9, 67.5, 67.4, 67.3, 67.0, 59.3, 59.0, 42.2, 42.0, 41.8, 41.6, 23.13, 23.08, 23.05, 22.97. ^1^H NMR (DMSO-d_6_, 400 MHz, 150 °C) δ 9.94 (br. s, 1H), 7.21 (dd, *J* = 7.8, 6.3 Hz, 1H), 6.69-6.58 (m, 5H), 6.41 (br. s, 2H), 5.88 (s, 4H), 4.75-4.71 (m, 2H), 4.68 (br. s, 1H), 4.65 (br. s, 1H), 3.76-3.57 (m, 6H), 2.07-1.83 (m, 4H), 1.70 (s, 3H), 1.68 (s, 3H); ^13^C NMR (DMSO-d_6_, 100 MHz, 150 °C) δ 179.5, 162.6 (d, ^1^*J*_C-F_ = 241 Hz), 152.7, 147.6, 143.5, 143.4, 143.1, 142.0, 130.7, 127.2, 127.1, 121.8, 112.1, 112.0, 108.8, 107.8, 107.5, 101.5, 97.8, 97.6, 97.2, 74.2, 68.3, 68.1, 59.6, 42.2, 41.4, 22.90, 22.85. HRMS [M+H]^+^ calcd. for C_34_H_35_FNO_9_ 620.2296, found 620.2294.

### Extraction and quantification of sterols

To quantify the abundance of sterols in *C. auris* the targeted metabolomics profiling protocol established by Hoepfner et al.^13^ in *S. cerevisiae* was used. Cells were subcultured to an OD_600_ of 0.1 in 10 mL of RPMI supplemented with the indicated compound concentration for 18 h with agitation. After incubation growth (OD_600_) was normalized and cell pellets washed and resuspended in 100 µL of PBS. Cell suspension was treated with 1 mL methanol/CHCl_3_ (2:1 v/v) supplemented with 0.01% w/v butylated hydroxytoluene. Acid washed glass beads were added to each sample and they were vortexed for 10 min. Samples were pelleted by centrifugation for 5 min at 16,000×*g*. Transferring the supernatant to a new vial, 400 μL 50 mM citric acid in H_2_O, and 600 μL CHCl_3_ was added and vortexed for 10 min. Samples were again centrifuged for 5 min at 16,000×g. The organic phase was collected and dried. For LC-MS analysis, samples were resuspended in ethanol with cholesterol included as an internal standard. Samples were separated on Acquity UPLC BEH C18 column (1.7 μm, 2.1 × 50 mm) using the Acquity UPLC I-Class coupled to a Xevo G2-S QToF equipped with an APCI source (Waters). Chromatographic methods were adopted from Hoepfner et al. as well as the selective reaction monitoring mass transitions specific for each sterol in subsequent quantification steps. TargetLynx (Waters; version 4.1) was used for peak finding, smoothing and area calculations. All samples were run in biological duplicate and technical triplicate, and a representative replicate was plotted in GraphPad Prism (version 8.4.2).

### Intracellular fluconazole and azoffluxin detection

*C. auris* was subcultured from overnight cultures at a starting OD_600_ of 0.4 in 5 mL of YPD in the presence of the indicated compound concentration for 1 h with agitation. Cells were then transferred to falcon tubes and pelleted at 3000×*g* for 5 min at 4 °C. Media was removed, and cells were washed with 5 mL of cold PBS three times with centrifugation of 2000×*g* for 5 min in between. Cells were resuspended in 1 mL cold PBS, flash frozen in liquid nitrogen, and stored at −80 °C overnight. The following day, cells were thawed on ice, 25 μL of 6 N NaOH was added to each falcon tube, and samples were vortexed for 15 s. In all, 500 μL of 10 mM sodium phosphate (pH 6.0) was added to each sample followed by vortexing for 15 s. Compounds were extracted with 5 mL of CH_2_Cl_2_ and vortexed for 5 min, followed by centrifugation for 10 min at 4000×*g* at 4 °C. The organic phase was collected and dried. Before subsequent LC-MS analysis, samples were resuspended in 50 μL MeCN:H_2_O. The resuspended cell extracts (10 µl) were separated on an Acquity UPLC BEH C18 column (1.7 μm, 2.1 × 50 mm) using the Acquity UPLC I-Class coupled to a Xevo G2-S QToF equipped with an electrospray ionization (ESI) source. Chromatography followed a gradient method (A: water + 0.1% (v/v) formic acid, B: MeCN + 0.1% (v/v) formic acid | 0–9 min: 10% B to 95% B at 0.125 µL min^−1^). Both fluconazole and azoffluxin were detected using selected reaction monitoring mass transitions 307.110 [M+H]^+^ → 220.0685 and 602.227 [M-H_2_O+H]^+^ → 286.0515, respectively. TargetLynx (Waters) was used for peak finding, smoothing and area calculations. All samples were run in biological duplicate and technical triplicate and a representative replicate was plotted in GraphPad Prism.

### Quantitative real-time-PCR

To determine changes in efflux gene expression, strains were subcultured from a saturated overnight culture at an OD_600_ 0.1 in YPD for 3 h in the presence of compound as indicated. Cells were then pelleted at 3000×*g* at 4 °C, washed with cold PBS, flash frozen with liquid nitrogen, and stored at −80 °C. Cells were lysed by bead beating 4x for 30 s with 1 min on ice in between. RNA was extracted from lysed cells using the QIAGEN RNeasy kit and DNase treated using the QIAGEN RNase free DNAase Set. Complementary DNA synthesis was performed using the iScript cDNA Synthesis Kit (Bio-Rad). Quantitative real-time-PCR (RT-qPCR) was performed using in a 384-well plate, with a 10 μL reaction volume using Fast SYBR Green Master Mix (Applied Biosystems) and the BioRad CFX-384 Real Time System with the following cycling conditions: 95 °C for 3 min, then 95 °C for 10 s and 60 °C for 30 s, for 40 cycles. The melt curve was completed with the following cycle conditions: 95 °C for 10 s and 65 °C for 10 s with an increase of 0.5 °C per cycle up to 95 °C. Reactions were performed in technical triplicate using the primer pairs: *CDR1* (oLC6125/oLC6126), *MDR1* (oLC8050/oLC8051), *CDR4*-1 (oLC8052/oLC8053), *CDR4*-2 (oLC8054/oLC8055), *SNQ2*-1 (oLC8060/oLC8057), *SNQ2*-2 (oLC8058/oLC8059), and normalized to the house keeping genes *ACT1* (oLC5727/oLC5728) and *GPD1* (oLC5729/oLC5730). Primer sequences are included in Supplementary Information. Data were analyzed using the BioRad CFX Manager 3.1. Error bars depict standard error of the means of technical triplicates, representing the data from one of two biological replicates.

### Nile red accumulation assay

Cellular efflux was determined by measuring Nile red accumulation^34^. Cells were subcultured from an OD_600_ 0.1 in YPD for 4 h until exponential phase was reached. For cells treated with azoffluxin, 50 μM was added for 10 min prior to a 20-min incubation of all stained cells with 7 μM (3.5 mM in DMSO stock) of Nile red (Sigma). Cells were then pelleted for 1 min at 3000×*g* and resuspended in PBS. To quantify fluorescence, a CytoFlex Flow Cytometer (Beckman Coulter) was used. Cells were added to flat bottom, transparent, 96-well plate (Beckman Coulter). Each sample was run using the CytExpert Software (version 2.4) until ~20,000 events had been recorded. Populations were gated to exclude debris and doublets, and the median PE value was taken for each sample (Fig. S3 and Supplementary Data 1). To visualize samples, PBS cell suspensions were imaged by differential interference contrast (DIC) microscopy and the DsRed channel on a Zeiss Axio Imager.MI (Carl Zeiss) at the same exposure time. All experiments were performed in biological triplicate and a Student’s *t* test was used to compare significant differences in the fold-change upon azoffluxin treatment between strains when indicated.

### Co-culture experiments

To assess the ability of azoffluxin to rescue mammalian cell growth in co-culture experiments, 20 μL of 293 T cells were seeded at 1 × 10^5^ cells/mL in DMEM media containing 10% FBS and incubated overnight at 37 °C in 5.5% CO_2_. The following day 20 μL of DMEM inoculated with 2.5 × 10^3^ cells/mL exponential phase *C. auris* cells was added to the wells. A Tecan D300e compound dispenser was used to add DMSO-based compounds to each well at the indicated final concentrations. Co-cultures were incubated for 48 h at 37 °C in 5.5% CO_2_. The mammalian cell growth was measured by replacing the media with 20 μL PBS, and adding 20 μL Titer-glow (Promega) to each well, incubating for 10 min, and reading luminescence on a Tecan Infinite 200 Pro. All experiments were performed in technical quadruplicate and biological duplicate and plotted in GraphPad Prism.

The cellular glycoproteins were stained in co-culture experiments using a periodic-acid Schiff (PAS) staining kit (Sigma) as per manufacturer’s instructions. Mammalian 293 T cells were seeded at 5 × 10^4^ cells/well for 24 h in six-well plates (Corning). In all, 2.5 × 10^3^ exponential phase *C. auris* cells were added to mammalian cells followed by indicated drug or solvent concentrations. Plates were incubated for 48 h at 37 °C. Cultures were fixed with 4% formaldehyde (BioShop) in medium overnight, fixative was removed, and the plate was dried. Fixed cells were then hydrated with 1 mL ddH_2_O and this was removed from each well. In total, 1 mL PAS solution was added and cells were incubated for 5 min, followed by removal and 2x washes with ddH_2_O by pipetting. In all, 1 mL Schiff’s reagent was added, and plates were incubated for 15 min. The cells were then thoroughly rinsed for 5 min with ddH_2_O. In all, 1 mL hematoxylin was applied for 3 min and cell were rinsed again. Cells were allowed to dry and were then imaged. Experiments were performed in biological duplicate, with one representative image being shown.

### Plasma stability assay

To assess the ability of azoffluxin to retain activity after exposure to mouse serum, a 10x concentration of azoffluxin was incubated for 1 h in 100% mouse plasma in sodium citrate buffer (Cedarlane, CL8001). This was incubated along with the control compounds gepinacin, which is known to be metabolized by serum, and caspofungin, which is not metabolized by serum. Compounds were incubated at either at 37 °C or on ice, or in the absence of serum in a YPD medium control at 37 °C. 10 μL of the mixture was then added to 90 μL of YPD inoculated with *C. auris* Ci6684 to a final concentration of ~1 × 10^3^ cells/mL in a 96-well plate. Plates were incubated at 30 °C and growth was measured after 48 h by OD_600_. All experiments were performed in technical triplicate and biological duplicate.

### Mouse studies

The pharmacokinetics of azoffluxin in mice were measured by the Preclinical Pharmacology Core at UT Southwestern Medical School. All animal work was approved and conducted under the oversight of the UT Southwestern Institutional Animal Care and Use Committee, which uses the Guide for the Care and Use of Laboratory Animals when establishing animal research standards. Mice were maintained at 20.5–22.2 °C, at 48–52% humidity, with light/dark alternating every 12 h. 21 6-week-old female CD-1 (Charles River) mice were dosed intraperitoneally (IP) with 10 mg/kg azoffluxin. Azoffluxin was formulated at 1 mg/mL in 25% PEG-400 (Sigma); 10% DMSO; 0.1% Tween-80 (Sigma), and 65% ddH_2_O. At the indicated times post dose (*n* = 3 per time point), mice were bled via a submandibular site and whole blood was collected in lavender top K_2_EDTA tubes. Plasma was processed from whole blood by centrifugation at 9600×*g* for 10 min. In total, 50 µL plasma was either used straight or was diluted in commercial K_2_EDTA CD-1 plasma (BioIVT) and mixed with 100 µL of methanol containing 0.15% formic acid, 3 mM Ammonium Acetate, and 37.5 ng/mL *N*-benzylbenzamide internal standard. The mixture was vortexed, incubated for 10 min at room temperature, and then centrifuged at 16,100 g for 5 min. Supernatant was collected and recentrifuged. The final supernatant was analyzed by LC-MS/MS. Analytical standards and quality control samples were prepared in a similar fashion by spiking commercial K_2_EDTA CD-1 mouse plasma with known quantities of azoffluxin. Chromatography conditions were as follows: an Agilent (Santa Clara) C18 XDB, 5 µm packing, 50 × 4.6 mm size column was used for reverse phase chromatography. Buffer A consisted of dH_2_O + 2 mM NH_4_ acetate and 0.1% formic acid and Buffer B consisted of methanol + 2 mM NH_4_ acetate and 0.1% formic acid. Gradient conditions utilized were: 0.01–0.5 min 3% B, 0.5–1.5 min gradient to 100% B,1.5–2.5 min 100% B, 2.5–2.6 min gradient to 3% B, 2.6–3.6 min 3% B. A 619.9 to 286.1 transition was used to quantitate azoffluxin while the 619.9 to 384.2 transition was used as the qualifier ion pair. N-benzylbenzamide (212.1 to 91.1 transition) was used as an internal standard. Data were analyzed using Analyst software (AB Sciex.; version 1.7.1) A value 3x above the signal obtained in the blank plasma was designated as the limit of detection (LOD). The limit of quantitation (LOQ) was defined as the lowest concentration on the standard curve at which back calculation yielded a concentration within 20% of the theoretical value and above the LOD signal. The LOQ for azoffluxin was 0.1 ng/ml. Pharmacokinetic properties were evaluated using the noncompartmental analysis tool in WinNonlin (Certara, Corp.; Phoenix WinNonlin version 8.1). Sparse sampling was used for data analysis. Terminal half-life was calculated as the ln(2)/λz where λz is a first order rate constant associated with the terminal (log-linear) portion of the curve. It is estimated by linear regression of time vs. log concentration by the software for three or more of the final nonzero data points. Tmax (time to maximal drug concentration) and Cmax (maximal drug concentration) were determined by visual inspection. Area under the concentration time curve (AUC_last_) from time 0 to the last observed concentration was determined by linear trapezoidal analysis. Apparent Volume of Distribution (Vz_F) is based on the terminal phase and is calculated as Dose/λz*AUC_inf__obs while Apparent Clearance (CL_F) is calculated as Dose/AUC_inf__obs. Calculation of Absolute Vz and CL requires knowledge of IP bioavailability (F) which is calculated as AUC_IP_/AUC_iv_ X Dose_iv_/Dose_IP_ but was not determined here because an iv PK was not performed. Mean residence time (MRT_last_) is the average time a molecule of drug spends in the system before before the last measurable concentration and is calculated by dividing the area under the curve formed by time and the product of concentration and time (AUMC) divided by the AUC.

The in vivo tolerability of azoffluxin was determined in outbred ICR (CD-1) mice (Envigo). Animal toxicity studies were approved by the Institutional Animal Care and Use Committee of The Lundquist Institute for Biomedical Innovation at Harbor-UCLA Medical Center (#31413), according to the NIH guidelines for animal housing and care. Mice were maintained at 20.5–22.2 °C, at 30–70% humidity, with light/dark alternating every 12 h. Mice weighing ~ 25 g were rendered neutropenic by administration of two doses of cyclophosphamide (200 mg/kg; Sandoz) given IP and cortisone acetate (250 mg/kg; Sigma-Aldrich) given subcutaneously on day −2 and +3, relative to treatment. To prevent bacterial infection, mice received antibacterial prophylaxis consisting of 50 μg/mL enrofloxacin (Bayer) in the drinking water starting the same day of immunosuppression. Mice were treated with 10 mg/kg of azoffluxin formulated as described above twice daily for 4 days or left untreated (*n* = 5). Mice were monitored twice daily for signs of distress such as hunching, ruffled fur, weight loss, difficulty moving, or reduced drinking or eating to prevent and minimize unnecessary pain for 21 days.

The antifungal activity of azoffluxin was assessed using a well-characterized model^49^. All animal procedures were approved by the Institutional Animal Care and Use Committee at the University of Wisconsin and William S Middleton VA according to guidelines of the Animal Welfare Act (#DA0081), The Institute of Laboratory Animal Resources Guide for the Care and Use of Laboratory Animals, and Public Health Service Policy. Mice were maintained at 22.2 °C, at 45% humidity, with light/dark alternating every 12 h. Specific-pathogen-free, six-week-old female ICR (CD-1) mice weighing 23 to 27 g were used (Envigo). Mice were rendered neutropenic by cyclophosphamide (Mead Johnson Pharmaceuticals) subcutaneous injection 4 days (150 mg/kg) and 1 day (100 mg/kg) before infection and 2 days after infection (100 mg/kg). *C. auris* isolate B11801was subcultured on SDA 24 h prior to infection. The inoculum was prepared by placing three to five colonies into 5 mL of sterile pyrogen-free 0.9% saline that had been warmed to 35 °C. The final inoculum was adjusted to a 0.6 transmittance at 530 nm. Final inoculum was determined to be 5.97 ± 0.03 log_10_ CFU/mL. Disseminated infection was achieved by injection of 0.1 mL of inoculum via the lateral tail vein 2 h prior to the start of drug therapy. Mice were treated with either saline as a control, fluconazole 32 mg/kg administered subcutaneously every 12 h, azoffluxin 10 mg/kg administered IP every 6 h, or fluconazole 32 mg/kg administered subcutaneously every 12 h and azoffluxin 10 mg/kg administered IP every 6 h. Animals were monitored every 6 h for signs of distress. Mice displaying hunching, ruffled fur, difficulty moving, or reduced drinking or eating were immediately euthanized. Mice were treated for 96 h, then sacrificed by CO_2_ asphyxiation. After sacrifice, the kidneys of each mouse were removed and placed in sterile 0.9% saline at 4 °C. Kidney homogenates were prepared and serially diluted 1:10. Aliquots were plated on SDA for viable fungal colony counts after incubation for 24 h at 35 °C. The lower limit of detection was 100 CFU/mL. Results were expressed as the mean number of CFU per kidney for three mice. Controls were sampled at the start and end of the 96-hour treatment period. These experiments were performed in two replicates.

### Reporting summary

Further information on research design is available in the Nature Research Reporting Summary linked to this article.

## Supplementary information

Supplementary Information

Description of Additional Supplementary Files

Supplementary Data 1

Reporting Summary

## Data Availability

Flow cytometry data collected for this study and its analysis is included as Supplementary Data 1. Additional data that supports the findings of this study are available from the corresponding author upon reasonable request. Source data are provided with this paper.

## Source data

Source Data
