# Supplementary material for: An oxindole efflux inhibitor potentiates azoles and impairs virulence in the fungal pathogen Candida auris
Source: Nat Commun. 2020 Dec 22;11:6429. doi: 10.1038/s41467-020-20183-3 (PMC7755909; doi:10.1038/s41467-020-20183-3)
Supplement: Supplementary file 1 — Supplementary Information [file 41467_2020_20183_MOESM1_ESM.pdf]

An oxindole efflux inhibitor potentiates azoles and impairs virulence in the fungal pathogen *Candida auris*.

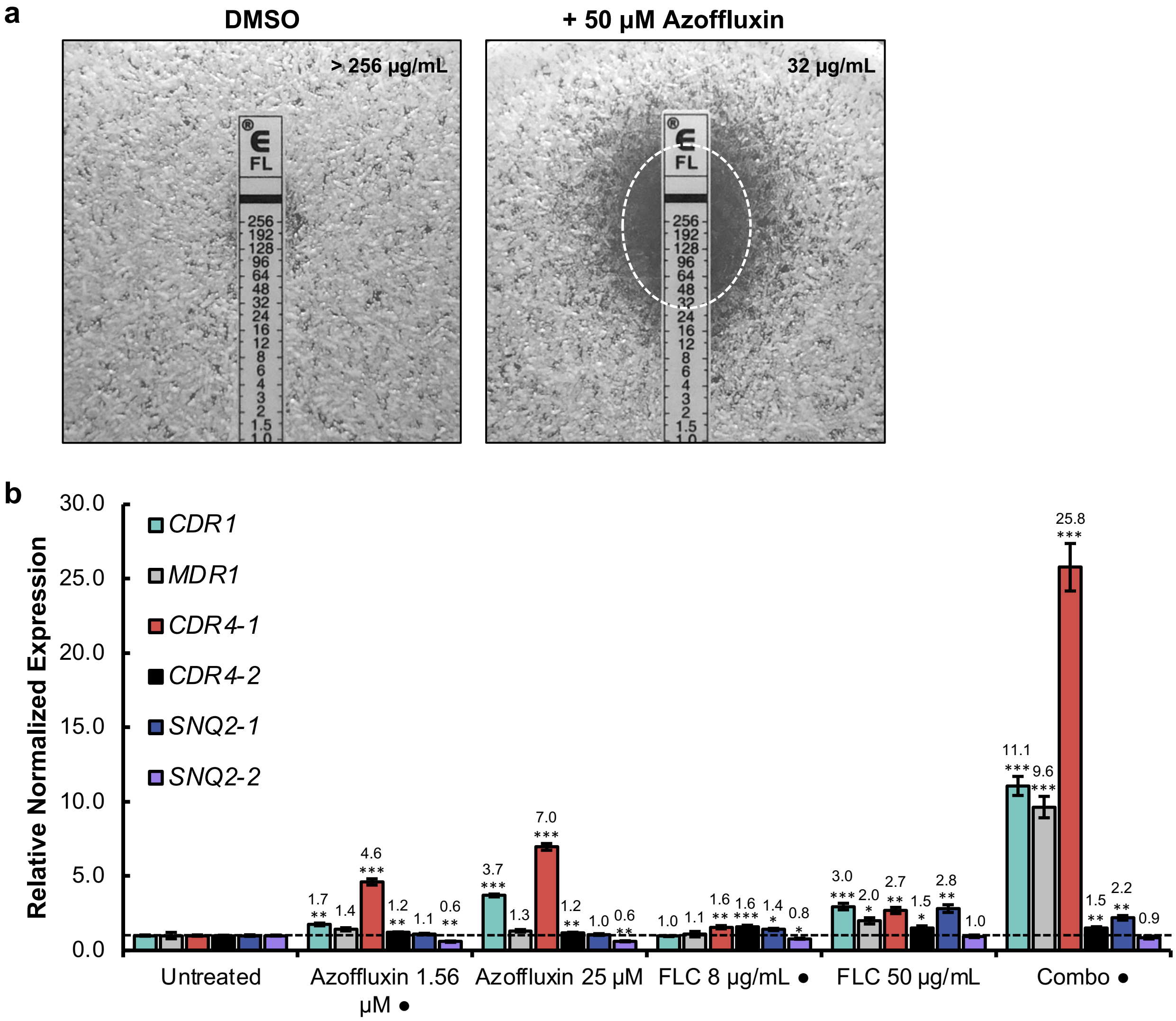

Fig. S1. Fluconazole (FLC) sensitivity and efflux gene expression upon azoffluxin treatment. a FLC Etest strips in the presence and absence of 50 µM azoffluxin. A total of 1×10^6 *C. auris* cells were spread onto YPD agar plates, the E-test strip was added, and plates were incubated at 30°C for 24 hours prior to imaging. The minimum inhibitory concentration is in the top right of each image and the dotted white line shows the zone of inhibition. **b** The relative transcript levels of a panel of putative efflux pumps in *C. auris* Ci6684 was quantified. *CDR1* (B9J08_000164; teal), *MDR1* (B9J08_003981; grey), *CDR4-1* (B9J08_000479; red), *CDR4-2* (B9J08_002451; black), *SNQ2-1* (B9J08_001125; blue), and *SNQ2-2* (B9J08_004452; purple) were measured. Cells were treated for 3 hours (• indicates concentrations of fluconazole (FLC) and azoffluxin used in combination (combo) treatment). Transcript levels were normalized to *ACT1* and *GPD1* and are plotted relative to the untreated control. Data are presented as mean +/- SEM of three technical triplicates. Significance of differences between respective untreated control and treatment was determined by two-sided unpaired student t-test; (*) p-value < 0.05, (**) p-value < 0.01, and (***) p-value < 0.001. Fold-change is indicated above each bar for the respective treatment. Source data are provided as a Source Data file.

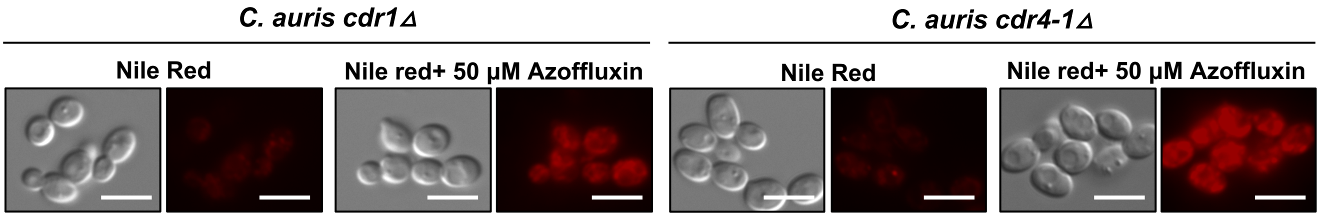

Fig. S2. **Azoffluxin increases Nile red accumulation in *C. auris cdr1Δ* and *cdr4-1Δ* strains.** *C. auris* Ci6684 strains with the efflux pumps *CDR1* and *CDR4-1* deleted were grown to exponential phase and treated with azoffluxin, followed by addition of Nile red. Cells were then imaged on the DIC and DsRed channels at equivalent exposure times as those in Fig. 2d. Micrographs represent two independent replicates. Scale bar represents 5 μm.

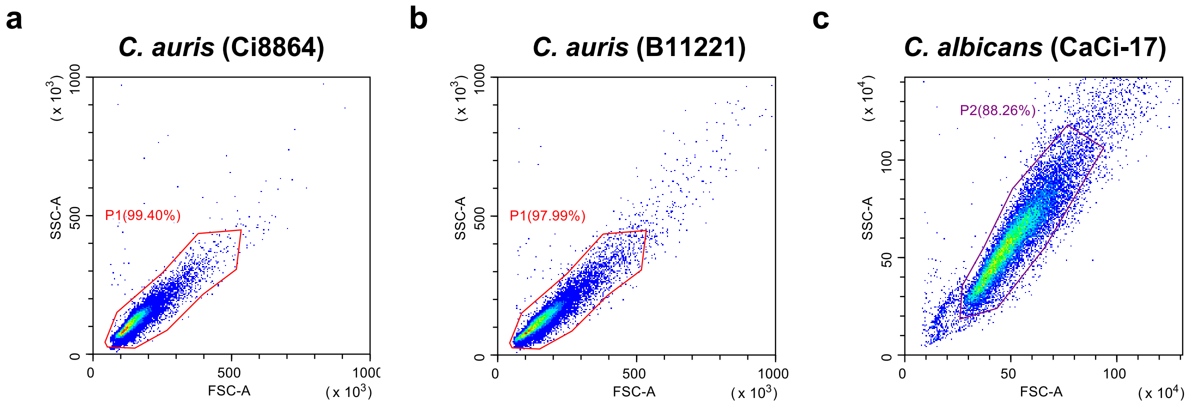

Fig. S3. Summary of gating strategy from Nile red accumulation assay. Pseudo-coloured density plots displaying the side-scatter and forward-scatter data for each even recorded in an untreated sample of *C. auris* and *C. albicans* cells when run on the Cytoflex flow cytometer and analyzed with the CytExpert Software. Gates displayed in red are the gates that were applied to all samples for data shown in panel Fig. 2e (panel a), Fig. 5b (panel b), and Fig. 6b (Panel c).

Supplementary Table 1: Strains used in this study

Strain ID	Description	Genotype	Source
CaLC79	*C. albicans* CaCi-2	Clinical isolate	[1]
CaLC91	*C. albicans* CaCi-17	Clinical isolate	[1]
ScLC151	*S. cerevisiae* BY4741	*MATa his3Δ1 leu2Δ0 met15Δ0 ura3Δ0*	[2]
CaLC239	*C. albicans* SN95	*arg4Δ/arg4Δ, his1Δ/his1Δ, URA3/ura3Δ::imm434 IRO1/iro1Δ::imm434*	[3]
CgLC1002	*C. glabrata* BG2	Clinical isolate	[4]
CauLC3438	*C. auris*	VPCI 673/P/12	Gift from Anuradha Chowdhary
CaLC4255	*C. albicans* SN152 *tac1Δ/tac1Δ*	*arg4Δ/arg4Δ, leu2Δ/leu2Δ::LEU2, his1Δ/his1Δ::HIS1, URA3/ura3Δ, IRO1/iro1Δ, tac1Δ/tac1Δ*	[5]
CauLC5083	*C. auris* Ci6684	Clinical isolate Clade I	[6]
CauLC5280	*C. auris* Ci6684 *cdr1Δ*	CauLC5083 + *cdr1Δ::NatMX*	[7]
CauLC5288	*C. auris* B11220	Clinical isolate Clade II	[8,9]
CauLC5289	*C. auris* B11109	Clinical isolate Clade I	[8,9]
CauLC5290	*C. auris* B11221	Clinical isolate Clade III	[8,9]
CauLC5291	*C. auris* B11222	Clinical isolate Clade III	[8,9]
CauLC5292	*C. auris* B11244	Clinical isolate Clade IV	[8,9]
CauLC5293	*C. auris* B11245	Clinical isolate Clade IV	[8,9]
CauLC5294	*C. auris* B8441	Clinical isolate Clade I	[8,9]
CauLC5295	*C. auris* B11098	Clinical isolate Clade I	[8,9]
CauLC5296	*C. auris* B11203	Clinical isolate Clade I	[8,9]
CauLC5297	*C. auris* B11205	Clinical isolate Clade I	[8,9]
CalC5447	*C. albicans* CaCi-17 *cdr1Δ /cdr1Δ*	Clinical isolate *cdr1Δ /cdr1Δ*	This study
CaLC5589	*C. albicans TAC1/TAC1*	CaLC4255 *(tac1::HIS1)::TAC1WT-SAT1/(tac1::LEU)::TAC1WT-HIS1*	This study
CaLC5591	*C. albicans* $TAC1^{M677\Delta}/TAC1^{M677\Delta}$	CaLC4255 *(tac1::HIS1)::TAC1 M677Δ-SAT1/(tac1::LEU2)::TAC1 M677Δ-HIS1*	This study
CaLC5593	*C. albicans* $TAC1^{N972D}/TAC1^{N972D}$	CaLC4255 *(tac1::HIS1)::TAC1 N972D-SAT1/(tac1::LEU2)::TAC1 N972D-HIS1*	This study
CaLC5595	*C. albicans* $TAC1^{N977D}/TAC1^{N977D}$	CaLC4255 *(tac1::HIS1)::TAC1 N977D-*	This study

		SAT1/(tac1::LEU2)::TAC1 N977D-HIS1	
CauLC6410	*C. auris cdr1Δ*	CauLC5083 + *cdr4-1Δ::NatMX*	This study
CauLC6554	*C. auris* B12037	Clinical isolate Clade III	Gift from Philippe Dufresne
CauLC6750	*C. auris* B12037 *cdr1Δ*	CauLC6654 + *cdr1Δ::NatMX*	This study
B11801	*C. auris* B11801	Clinical isolate Clade IV	[9]
HEK 293T	HEK 293T	Firefly luciferase expressing	[10]

Supplementary Table 2: Plasmids used in this study

Plasmid ID	Description	Source
pLC1049	*C. auris NATMX* marker	[7]
pLC1081	*C. albicans CAS9* vector pV1093	[11,12]
pLC1083	*C. albicans CDR1-SAT1* flipper	[12]
pLC1092	*C. albicans TAC1* vector pFA-TAC1-HIS-T3)	[13]
pLC1093	*C. albicans* $TAC1^{M677\Delta}$ vector pFA-TAC1 M677Δ-HIS-T3	[13]
pLC1094	*C. albicans* $TAC1^{N972D}$ vector pFA-TAC1N972D-HIS-T3	[13]
pLC1095	*C. albicans* $TAC1^{N977D}$ vector pFA-TAC1N977D-HIS-T3	[13]

Supplementary Table 3: Oligonucleotides used in this study

Oligo ID	Description	Sequence (5' to 3')
oLC274	pJK863down-F	CTGTCAAGGAGGGTATTCTGG
oLC1096	CaCDC37+505+SacI-R	CCCGAGCTCCGTCGATCCTGTTTCTATGT
oLC1097	CaCDC37+662-R	GGAGCTTTTGGTTTATCTTG
oLC1098	6xHIS-CaCTA8+1814+Apa1-F	TTGCGGGCCCGCAATTAGTTGGTCATCAACC
oLC1099	6xHIS-CaCTA8+2286+Apa1-R	TTGCGGGCCCTTAGTGGTGGTGGTGGTGGTGATGATCGCTAACTTCTTCG
oLC5727	CaurisACT1+121F	ACCCCAAGTCCAACAGAGAG
oLC5728	CaurisACT1+316R	TCCAGCCAAGTCAAGTCTCA
oLC5729	CaurisGPD1+141F	ATCCTTGCTGAAAACGCTGC
oLC5730	CaurisGPD1+318R	TCCTCGGCCACCTTTACAAT
oLC6020	CaurisCDR1-989F	TAACGCAAAAGGACCATGGC
oLC6023	CaurisCDR1+5493R	CGCCCTTGATAATGTCCACG
oLC6024	CaurisCDR1-925F	CGGCCCATGATAACCCTCTA
oLC6025	CaurisCDR1+5426R	TTTCTGTCTCTCTGAGGGCA
oLC6125	CauCDR1+3359F	CGCTGAATGGATGTTGGAGG
oLC6126	CauCDR1+3514R	CTTCTTTCTGGACTCCGGGT
oLC6169	CaurisCDR1+2679R	GCAGTGATCTGACCTGGCTT
oLC6221	CaurisCDR1-1298F	ACAGCTGGATTCGACATGGG
oLC6231	CaurisCDR1+2175F	TTTGTGCCTTCAGGAGGACC
oLC6296	pLC605 NAT F	ACTGGATGGCGGCGTTAGTA
oLC6304	pLC605 NAT R	ATCAAGCTTGCCTCGTCC

oLC6305	NAT_CaurisCDR1-35R	CTATACTGCTGTCGATTCGATACTAACGCCG CCATCCAGTACTACATGCGATATATATAT
oLC6306	NAT_CaurisCDR1+69F	CGCTGGCCGGGTGACCCGGCGGGGACGAGG CAAGCTTGATTGAGCTCGTGTGTGTCATCA
oLC6307	CaurisCDR1-797R	CCCACATTTCGAGAAAAGGA
oLC6308	pLC605NAT_26	TGGTCGCTATACTGCTGTCG
oLC6926	SNR52/F	AAGAAAGAAAGAAAACCAGGAGTGAA
oLC6927	sgRNA/R	ACAAATATTTAAACTCGGGACCTGG
oLC6928	SNR52/N	GCGGCCGCAAGTGATTAGACT
oLC6929	sgRNA/N	GCAGCTCAGTGATTAAGAGTAAAGATGG
oLC6966	SNR52/R_CDR1	ATACAAGTGAAAACATTCAGCAAATTAAAA ATAGTTTACGCAAGTC
oLC6967	sgRNA/F_CDR1	CTGAATGTTTTCACTTGTATGTTTTAGAGCT AGAAATAGCAAGTTAAA
oLC6968	sCdr1 NQ sense	ATTCTAAGATGTCGTCGCAAGATG
oLC6969	sCdr1 NQ anti	AGTTCTGGCTAAATTCTGAATGTTTTC
oLC7041	oLC7041_Tac1 check sense 1	TAAATGCAATGGGTCTTATCCATGTGG
oLC7042	oLC7042_Tac1 check anti 1	GTCAAATATTCTTCACCGTATGAACCT
oLC8050	CauMDR1orf_21 F	AGAGAGAGCTTCTTCGGCAG
oLC8051	CauMDR1orf_174 R	AGATCAACGGGGGTGTCTGA
oLC8052	CauCDR4_942orf_2102 F	TCCAGAACTGGGCAATAGCG
oLC8053	CauCDR4_942orf_2286 R	TGCATGGCTCCCTTGTTGAT
oLC8054	CauCDR4_069orf_1447 F	TTCGACTCAAAGGTGACCCG
oLC8055	CauCDR4_069orf_1596 R	AGGGAGCAGAACGCATTGAA
oLC8056	CauSNQ2_644orf_2600 F	ACCTGGTAAGTTGACCGCCT
oLC8057	CauSNQ2_644orf_2790 R	CGAAGGGCTTCTCTGACAGT
oLC8058	CauSNQ2_421orf_2042 F	CACTGTTTGTGGCTTCACCG
oLC8059	CauSNQ2_421orf_2210 R	CGCCTGAAACAGGTCTCACT
oLC8164	Cau_CDR4_942orf-943 F	GGATTTGGGATTTGGACACT
oLC8165	NAT_CauCDR4_942orf-5R	CTATACTGCTGTCGATTCGATACTAACGCCG CCATCCAGTGGTCCTGAGAAGTCGTGGAC
oLC8166	NAT_CaurisCDR4_942+33F	CGCTGGCCGGGTGACCCGGCGGGGACGAGG CAAGCTTGATCCACGGTAAAAACGATGGAC
oLC8167	Cauris_CDR4_942orf+965 R	ACCAGGCTTGGAATTGACAG
oLC8168	Cauris_CDR4_942orf-880 F	GCTGTGAGAGTTGGCAAGG
oLC8169	Cauris_CDR4_942orf+863 R	GCCAAATTCGCCATTAAAGA

Supplementary References

1. White, T. C. Increased mRNA levels of *ERG16*, *CDR*, and *MDR1* correlate with increases in azole resistance in *Candida albicans* isolates from a patient infected with human immunodeficiency virus. *Antimicrob. Agents Chemother.* **41**, 1482–1487 (1997).

2. Giaever, G. *et al.* Functional profiling of the *Saccharomyces cerevisiae* genome. *Nature* **418**, 387–391 (2002).

3. Noble, S. M. & Johnson, A. D. Strains and strategies for large-scale gene deletion studies of the diploid human fungal pathogen *Candida albicans*. *Eukaryot. Cell* **4**, 298–309 (2005).

4. Cormack, B. P. & Falkow, S. Efficient homologous and illegitimate recombination in the opportunistic yeast pathogen *Candida glabrata*. *Genetics* **151**, 979–87 (1999).

5. Homann, O. R., Dea, J., Noble, S. M. & Johnson, A. D. A phenotypic profile of the Candida albicans regulatory network. *PLoS Genet.* **5**, e1000783 (2009).

6. Chatterjee, S. *et al.* Draft genome of a commonly misdiagnosed multidrug resistant pathogen *Candida auris*. *BMC Genomics* **16**, 686 (2015).

7. Kim, S. H. *et al.* Genetic analysis of *Candida auris* implicates Hsp90 in morphogenesis and azole tolerance and Cdr1 in azole resistance. *mBio* **10**, e02529-18 (2019).

8. Lockhart, S. R. *et al.* Simultaneous emergence of multidrug-resistant *Candida auris* on 3 continents confirmed by whole-genome sequencing and epidemiological analyses. *Clin. Infect. Dis.* **64**, 134–140 (2017).

9. CDC & FDA Antibiotic Resistance Isolate Bank. Atlanta (GA):CDC (2020)

10. Stone, S. D., Lajkiewicz, N. J., Whitesell, L., Hilmy, A. & Porco, J. A. Biomimetic kinetic resolution: Highly enantio- and diastereoselective transfer hydrogenation of aglain ketones to access flavagline natural products. *J. Am. Chem. Soc.* **137**, 525–530 (2015).

11. Min, K., Ichikawa, Y., Woolford, C. A. & Mitchell, A. P. *Candida albicans* gene deletion with a transient CRISPR-Cas9 system. *mSphere* **1**, 1–9 (2016).

12. Liu, Z. & Myers, L. C. Mediator tail module is required for Tac1-activated *CDR1*

expression and azole resistance in *Candida albicans*. *Antimicrob. Agents Chemother.* **61**, 1–20 (2017).

13. Liu, Z., Rossi, J. M. & Myers, L. C. *Candida albicans* Zn cluster transcription factors Tac1 and Znc1 are activated by farnesol to upregulate a transcriptional program including the multidrug efflux pump *CDR1*. *Antimicrob. Agents Chemother.* **62**, e00968-18 (2018).
