# Supplementary material for: An oxindole efflux inhibitor potentiates azoles and impairs virulence in the fungal pathogen Candida auris
Source: Nat Commun. 2020 Dec 22;11:6429. doi: 10.1038/s41467-020-20183-3 (PMC7755909; doi:10.1038/s41467-020-20183-3)
Supplement: Supplementary file 2 — Description of Additional Supplementary Files [file 41467_2020_20183_MOESM2_ESM.pdf]

Description of Additional Supplementary Files

File Name: Supplementary Data 1
Description: Raw flow cytometry data obtained for this study.
