# Supplementary material for: An oxindole efflux inhibitor potentiates azoles and impairs virulence in the fungal pathogen Candida auris
Source: Nat Commun. 2020 Dec 22;11:6429. doi: 10.1038/s41467-020-20183-3 (PMC7755909; doi:10.1038/s41467-020-20183-3)
Supplement: Supplementary file 4 — Reporting Summary [file 41467_2020_20183_MOESM4_ESM.pdf]

nature research

Corresponding author(s): Leah E. Cowen

Last updated by author(s): November 3, 2020

Reporting Summary

Nature Research wishes to improve the reproducibility of the work that we publish. This form provides structure for consistency and transparency in reporting. For further information on Nature Research policies, see Authors & Referees and the Editorial Policy Checklist .

Statistics

For all statistical analyses, confirm that the following items are present in the figure legend, table legend, main text, or Methods section.

n/a	Confirmed	
☐	☒	The exact sample size (*n*) for each experimental group/condition, given as a discrete number and unit of measurement
☐	☒	A statement on whether measurements were taken from distinct samples or whether the same sample was measured repeatedly
☐	☒	The statistical test(s) used AND whether they are one- or two-sided<br>*Only common tests should be described solely by name; describe more complex techniques in the Methods section.*
☒	☐	A description of all covariates tested
☒	☐	A description of any assumptions or corrections, such as tests of normality and adjustment for multiple comparisons
☐	☒	A full description of the statistical parameters including central tendency (e.g. means) or other basic estimates (e.g. regression coefficient) AND variation (e.g. standard deviation) or associated estimates of uncertainty (e.g. confidence intervals)
☐	☒	For null hypothesis testing, the test statistic (e.g. *F*, *t*, *r*) with confidence intervals, effect sizes, degrees of freedom and *P* value noted<br>*Give P values as exact values whenever suitable.*
☒	☐	For Bayesian analysis, information on the choice of priors and Markov chain Monte Carlo settings
☒	☐	For hierarchical and complex designs, identification of the appropriate level for tests and full reporting of outcomes
☒	☐	Estimates of effect sizes (e.g. Cohen's *d*, Pearson's *r*), indicating how they were calculated

Our web collection on statistics for biologists contains articles on many of the points above.

Software and code

Policy information about availability of computer code

Data collection	TargetLynx (Waters; version 4.1) was used for peak finding, smoothing and area calculations in LC-MS experiments. Flow cytometry data was collected with the Beckman Coulter CytExpert Software (version 2.4).
Data analysis	BioRad CFX Manager (version 3.1) was used to normalize RT-qPCR data and plot mean values with calculated SEM. Excel (version 16.36) was used to plot bar graphs of RT-qPCR data, screening data, and calculate all statistical significance using a standard two-tailed unpaired student t-test. Flow cytometry data was analyzed with the Beckman Coulter CytExpert Software (version 2.4). Pharmacokinetic properties were evaluated using Analyst software (AB Sciex.; version 1.7.1) and the noncompartmental analysis tool in WinNonlin (Certara, Corp., St. Louis, MO; Phoenix WinNonlin version 8.1). GraphPad Prism (version 8.4.2) was used to generate all bar plots with data points overlayed and LC-MS dot plots. Java TreView (version 1.1.6r4) was used to generate all heat plots.

For manuscripts utilizing custom algorithms or software that are central to the research but not yet described in published literature, software must be made available to editors/reviewers. We strongly encourage code deposition in a community repository (e.g. GitHub). See the Nature Research guidelines for submitting code & software for further information.

Data

Policy information about availability of data

All manuscripts must include a data availability statement. This statement should provide the following information, where applicable:

- Accession codes, unique identifiers, or web links for publicly available datasets
- A list of figures that have associated raw data
- A description of any restrictions on data availability

Flow cytometry data collected for this study and its analysis is included as Supplementary Data 1. All raw data used to generate main manuscript and supplementary figures can be found in the Source Data file provided with this paper. Additional data that supports the findings of this study are available from the corresponding author upon reasonable request.

Field-specific reporting

Please select the one below that is the best fit for your research. If you are not sure, read the appropriate sections before making your selection.

[x] Life sciences [ ] Behavioural & social sciences [ ] Ecological, evolutionary & environmental sciences

For a reference copy of the document with all sections, see nature.com/documents/nr-reporting-summary-flat.pdf

Life sciences study design

All studies must disclose on these points even when the disclosure is negative.

Sample size	Sample size was always n=3 or greater when statistical analysis was required. All experiments were performed in biological duplicate or greater with little deviation between replicates.
Data exclusions	In flow cytometry experiments, events were excluded from calculation of median fluorescence intensity (MFI) by gating on forward and side scatter parameters to eliminate debris and multi-cell clumps that would skew data. Gating removed less than 15% of all acquired events. No other data were excluded from analysis.
Replication	In cases of fungal growth assessment, assays were performed in technical duplicates which were averaged, in all other cases experiments were performed in technical triplicate as indicated in figure legends and methods. Each experiment was performed in at least biological duplicate with both replicates showing similar results.
Randomization	Randomization was not relevant to the type of experimentation reported. All assays had a quantitative output, rather than qualitative, and therefore, randomization was not required to eliminate user bias.
Blinding	Blinding was not relevant to this study as this was not an observational study with no opportunity for bias to factor into quantitative results. All assays had a quantitative output, rather than qualitative, and therefore, blinding was not required to eliminate user bias.

Reporting for specific materials, systems and methods

We require information from authors about some types of materials, experimental systems and methods used in many studies. Here, indicate whether each material, system or method listed is relevant to your study. If you are not sure if a list item applies to your research, read the appropriate section before selecting a response.

Materials & experimental systems

n/a	Involved in the study	
[x]	[ ]	Antibodies
[ ]	[x]	Eukaryotic cell lines
[x]	[ ]	Palaeontology
[ ]	[x]	Animals and other organisms
[x]	[ ]	Human research participants
[x]	[ ]	Clinical data

Methods

n/a	Involved in the study	
[x]	[ ]	ChIP-seq
[ ]	[x]	Flow cytometry
[x]	[ ]	MRI-based neuroimaging

Eukaryotic cell lines

Policy information about cell lines

Cell line source(s)	Human HEK293T cells were obtained from American Type Culture collection (ATCC Cat# CRL-3216).
Authentication	Cell line was not authenticated as specific tissue of origin was non-critical to validity of results reported.
Mycoplasma contamination	All cell lines tested negative for mycoplasma contamination by PCR-based detection.
Commonly misidentified lines (See ICLAC register)	No commonly misidentified cell lines were used.

Animals and other organisms

Policy information about studies involving animals; ARRIVE guidelines recommended for reporting animal research

Laboratory animals	For pharmacokinetic experiments, 6-week old female CD-1 mice were used. For toxicity and infection studies, 6-week old female ICR(CD-1)mice were used.

Wild animals	No wild animals were used in this study.
Field-collected samples	No field-collected samples were used in this study.
Ethics oversight	The pharmacokinetic studies were approved and conducted under the oversight of the UT Southwestern Institutional Animal Care and Use Committee. The toxicity studies were approved by the Institutional Animal Care and Use Committee of The Lundquist Institute for Biomedical Innovation at Harbor-UCLA Medical Center (#31413), according to the NIH guidelines for animal housing and care. The infection studies were approved by the Institutional Animal Care and Use Committee at the University of Wisconsin and William S Middleton VA according to the guidelines of the Animal Welfare Act (#DA0081), The Institute of Laboratory Animal Resources Guide for the Care and Use of Laboratory Animals, and Public Health Service Policy.

Note that full information on the approval of the study protocol must also be provided in the manuscript.

Flow Cytometry

Plots

Confirm that:

- [x] The axis labels state the marker and fluorochrome used (e.g. CD4-FITC).
- [x] The axis scales are clearly visible. Include numbers along axes only for bottom left plot of group (a 'group' is an analysis of identical markers).
- [x] All plots are contour plots with outliers or pseudocolor plots.
- [x] A numerical value for number of cells or percentage (with statistics) is provided.

Methodology

Sample preparation	Cells were subcultured to exponential phase and then treated as indicated for 10 minutes, followed by the addition of 7 mM Nile red for 20 minutes. Cell were washed and resuspended in PBS to a density of ~0.1 OD and run in plate format on the flow cytometer.
Instrument	Beckman Coulter CytoFlex Flow Cytometer - CytoFLEX S BC21021
Software	CytExpert Software
Cell population abundance	Populations were gated to remove debris and clumps for calculation of MFI. Populations reported represent >85% of total events.
Gating strategy	Gating was performed on the basis of forward and side scatter parameters to define a uniform single cell population and eliminate debris and multi-cell clumps that would skew data. Gated populations comprised >85% of all events acquired for all experiments.

- [x] Tick this box to confirm that a figure exemplifying the gating strategy is provided in the Supplementary Information.
